# Runge–Kutta approximation for $C_0$-semigroups in the graph norm with applications to time domain boundary integral equations

**DOI:** 10.1007/s42985-020-00051-x

**Published:** 2020-11-24

**Authors:** Alexander Rieder, Francisco-Javier Sayas, Jens Markus Melenk

**Affiliations:** 1 Fakultät für Mathematik, Universität Wien, Vienna, Austria; 2 Department of Mathematical Sciences, University of Delaware, Newark, DE USA; 3 Institut für Analysis und Scientific Computing, Technische Universität Wien, Vienna, Austria

## Abstract

We consider the approximation of an abstract evolution problem with inhomogeneous side constraint using *A*-stable Runge–Kutta methods. We derive a priori estimates in norms other than the underlying Banach space. Most notably, we derive estimates in the graph norm of the generator. These results are used to study convolution quadrature based discretizations of a wave scattering and a heat conduction problem.

## Introduction

Many time dependent partial differential equations can be conveniently described in the language of strongly continuous semigroups. In this language, these initial boundary value problems resemble systems of ordinary differential equations, which suggests that they are amendable to the standard discretization schemes of multistep or Runge–Kutta type. Unlike the ODE case, one needs to pay special attention to the boundary conditions imposed by the generator of the semigroup. This, in most cases, leads to a reduction of order phenomenon, meaning that the convergence rates are (mainly) determined by the stage order of the Runge–Kutta method instead of the classical order. The a priori convergence of Runge–Kutta methods for semigroups has been extensively studied in the literature. Starting with the early works [8, 9], it has been established that conditions of the form $u(t) \in {\text {dom}}(A^{\mu })$, where $A$ is the generator of the semigroup and ${\text {dom}}$ denotes the domain of an operator, determine the convergence rates. In [25], this has been generalized to the case of non-integer $\mu \ge 1$ using the theory of interpolation spaces. Finally, in [1], the case of $\mu \in [0,1]$ was adressed, which is the case needed for PDEs with inhomogeneous boundary conditions. We point out that in the case of analytic semigroups, Lubich and Ostermann [19] had already established convergence also for inhomogeneous boundary conditions. All of these works focus on establishing convergence rates with respect to the norm of the underlying Banach space. In many applications one needs to establish convergence with respect to other norms, for example, in order to be able to bound boundary traces of the solution. Most notably, one might be interested in convergence of $A_{\star }u$, where $A_{\star }$ is an extension of the generator that disregards boundary conditions. If *u* is assumed to be in ${\text {dom}}(A)$, we get $A_{\star }u=Au$ and the convergence result can be easily established by using the fact that the time evolution commutes with the generator of the underlying semigroup (both in the continuous and discrete settings). If the boundary conditions are inhomogeneous, such a strategy cannot be pursued. It is the goal of this paper to establish convergence results for $A_{\star }u$ also for the case $u(t) \in {\text {dom}}(A^{\mu })$ for $\mu \in [0,1]$, again using the theory of interpolation spaces.

Similarly it is sometimes useful to compute discrete integrals of the time evolution by reusing the same Runge–Kutta method. Also in this case, we establish rigorous convergence rates.

Our interest in such estimates originally arose from the study of time domain boundary integral equations and their discretization using convolution quadrature (CQ). It has already been noticed in the early works (see e.g. [19]) that such discretizations have a strong relation to the Runge–Kutta approximation of the underlying semigroup. This approach of studying TDBIEs in a strictly time-domain way has recently garnered a lot of interest, see [3, 13, 15] and the monograph [31], as it potentially allows sharper bounds than the more standard Laplace domain based approach. Similar techniques have even been extended to the case of certain nonlinear problems in [4]. This paper can be seen as our latest addition to this effort. While the convergence rates provided by the Laplace-domain approach in [2] and the results in this current paper are essentially the same, the present new approach provides better insight into the dependence on the end-time of the computation (quadratic vs. general unknown polynomial behavior). This suggest that the present approach might be better suited for analyzing long term computations. It also fits more naturally with the time-domain analysis of the continuous problem and space discretization, as for example presented in [13].

The paper is structured as follows. Section 2 introduces the abstract setting and fixes notation, most notably for working with Runge–Kutta methods. Section 3 then contains the main estimates. Starting by summarizing known results from [1] in Sect. 3.1, we then formulate the main new results of this article in Sect. 3.2. After proving some preparatory lemmas related to Runge–Kutta methods in Sects. 4 and 5, we provide the proofs of the main estimates in Sect. 6. In Sect. 7, we show how our setting simplifies if we restrict our view to a subclass of admissible operators. In Sect. 8, to showcase how the theory developed in this paper is useful for this class of problems, we consider a simple exterior scattering problem in Sect. 8.3 and a heat transmission problem in Sect. 8.5. We note that Sect. 8.3 showcases the need for the bound on the discrete integral of the result, whereas Sect. 8.5 was chosen because, in order to bound the main quantity of interest on the boundary, we need to apply a trace theorem. This necessitates the use of the graph norm estimate.

## Problem setting

We start by fixing the general setting used for the rest of the paper, first with respect to the equation to be solved and then with respect to its discretization.

### Operator equation, functional calculus, and Sobolev towers

#### Assumption 2.I

We are given:

- A closed linear operator $A_{\star }:{\text {dom}}\left(A_{\star }\right)\subset {\mathcal {X}} \rightarrow {\mathcal {X}}$ in a Banach space ${\mathcal {X}}$.
- A bounded linear operator $B: {\text {dom}} \left(A_{\star }\right)\rightarrow {\mathcal {M}}$ with another Banach space ${\mathcal {M}}$.

We assume that $A:=A_{\star }|_{\ker {B}}$ generates a $C_0$-semigroup and that *B* admits a bounded right inverse ${\mathscr {E}}$ such that $\mathrm {range}\,{\mathscr {E}}\subset \ker (I-A_{\star })$, where $I:{\mathcal {X}}\rightarrow {\mathcal {X}}$ is the identity operator.

We are given $u_0\in {\text {dom}} (A)$ and data functions $F \in {{\mathcal {C}}}^1([0,T], {\mathcal {X}})$, $\varXi \in {\mathcal {C}}^1([0,T],{\mathcal {M}})$, and we consider the problem: find $u \in {{\mathcal {C}}}^1([0,T],{\mathcal {X}})$ such that

2.1a $$\begin{aligned} {\dot{u}}(t)&= A_{\star }u(t) + F(t), \quad t>0, \end{aligned}$$

2.1b $$\begin{aligned} B u(t)&=\varXi (t), \quad t> 0, \end{aligned}$$

2.1c $$\begin{aligned} u(0)&=u_0. \end{aligned}$$

For conditions on the well-posedness of this problem, see [13]. We start by recalling the following consequence of the Hille-Yosida theorem.

#### Proposition 2.1

([26, Corollary 3.8]) If *A* is the generator of a $C_0$-semigroup on a Banach space ${\mathcal {X}}$, then there exist constants $\omega \ge 0$ and $M\ge 1$ such that the spectrum $\sigma (A)$ of *A* satisfies $\sigma (A) \subseteq \{z \in {\mathbb {C}}: \mathrm {Re}\,z \le \omega \}$ and the resolvent satisfies the estimates

2.2 $$\begin{aligned} \left\| \left( A-z I\right) ^{-1}\right\| _{{\mathcal {X}} \rightarrow {\mathcal {X}}}\le \frac{M}{ \mathrm {Re}\,z - \omega } \quad \forall z \text{ s.t. } \mathrm {Re}\,z>\omega . \end{aligned}$$

When working with Runge–Kutta methods, it is useful to use a calculus that allows one to apply rational functions to (unbounded) operators, as long as the poles of the function are compatible with the spectrum of the operator.

#### Definition 2.2

*(Rational functions of operators)* Let *q* be a rational function that is bounded at infinity. Let $\varLambda$ be the set of poles of *q*, which we can write in the form (note that we allow for some of the factors in the numerator to be constant)

$$\begin{aligned} q(z)=c_0 \prod _{i=1}^n \frac{c_i z-1}{z-\lambda _i} =c_0\prod _{i=1}^n \left( c_i+\frac{c_i\lambda _i-1}{z-\lambda _i}\right) . \end{aligned}$$

If $A:{\text {dom}}(A)\subset {\mathcal {X}}\rightarrow {\mathcal {X}}$ is a linear operator such that $\sigma (A)\cap \varLambda =\emptyset$, we define

2.3 $$\begin{aligned} q(A):= c_0 (c_1I+(c_1\lambda _1-1)(A-\lambda _1 I)^{-1}) \cdots (c_nI+(c_n\lambda _n-1)(A-\lambda _n I)^{-1}). \end{aligned}$$

It is easy to see that different reorderings of the factors in the numerator and denominator of *q* produce the same result and that each factor in the definition of *q*(*A*) is a bounded linear operator in ${\mathcal {X}}$ since $\lambda _i\not \in \sigma (A).$ The bounded linear operator $q(A):{\mathcal {X}} \rightarrow {\mathcal {X}}$ satisfies

2.4 $$\begin{aligned} \Vert q(A)\Vert _{{\mathcal {X}} \rightarrow {\mathcal {X}}} \le C_q \Big (1+ \big ( \max _{\lambda \in \varLambda }\Vert (A-\lambda I)^{-1}\Vert _{{\mathcal {X}}\rightarrow {\mathcal {X}}} \big )^n\Big ). \end{aligned}$$

The error estimates of this paper use the theory of interpolation spaces. For Banach spaces ${\mathcal {X}}_1 \subset {\mathcal {X}}_0$ with continuous embedding and $\mu \in (0,1)$, we define the space $[{\mathcal {X}}_0,{\mathcal {X}}_1]_{\mu ,\infty }$ using real interpolation with the following norm:

2.5 $$\begin{aligned} \left\| u\right\| _{[{\mathcal {X}}_0,{\mathcal {X}}_1]_{\mu ,\infty }}&:={\text {ess sup}}_{t>0}{\left( t^{-\mu } \inf _{v \in {\mathcal {X}}_1} \left[ \left\| u-v\right\| _{{\mathcal {X}}_0} + t \left\| v\right\| _{{\mathcal {X}}_1}\right] \right) }. \end{aligned}$$

We will not go into details of the definitions and instead refer to [35, 36] or [22, Appendix B]. For simplicity of notation we often drop the second parameter $\infty$ and just write $[{\mathcal {X}}_0,{\mathcal {X}}_1]_{\mu }$.

The most important property is the following: a bounded linear operator $T: {\mathcal {X}}_0 \rightarrow {\mathcal {Y}}_0$ and ${\mathcal {X}}_1 \rightarrow {\mathcal {Y}}_1$ with ${\mathcal {X}}_1 \subseteq {\mathcal {X}}_0$ and ${\mathcal {Y}}_1 \subseteq {\mathcal {Y}}_0$ is also a bounded operator mapping $[{\mathcal {X}}_0,{\mathcal {X}}_1]_{\mu } \rightarrow [{\mathcal {Y}}_0,{\mathcal {Y}}_1]_{\mu }$ with the following norm bound

2.6 $$\begin{aligned} \left\| T\right\| _{[{\mathcal {X}}_0,{\mathcal {X}}_1]_{\mu } \rightarrow [{\mathcal {Y}}_0,{\mathcal {Y}}_1]_{\mu }} \le \left\| T\right\| _{{\mathcal {X}}_0 \rightarrow {\mathcal {Y}}_0}^{1-\mu } \left\| T\right\| _{{\mathcal {X}}_1 \rightarrow {\mathcal {Y}}_1}^{\mu }. \end{aligned}$$

We also note that for $\mu _1 \le \mu _2$, the spaces are nested, i.e., $[{\mathcal {X}}_0,{\mathcal {X}}_1]_{\mu _2} \subseteq [{\mathcal {X}}_0,{\mathcal {X}}_1]_{\mu _1}$ with continuous embedding. For notational convenience we write $[{\mathcal {X}}_0,{\mathcal {X}}_1]_{0}:={\mathcal {X}}_0$ and $[{\mathcal {X}}_0,{\mathcal {X}}_1]_{1}:={\mathcal {X}}_1$. We will be interested in a collection of spaces defined by interpolating the domains of the powers of the operator *A*. The details of this construction can be found, for example in [11].

#### Definition 2.3

*(Sobolev towers)* Let $A$ be a closed operator on a Banach space ${\mathcal {X}}$. For $\mu \in {\mathbb {N}}_0$, we define the following spaces ${\mathcal {X}}_0:={\text {dom}} \left(A^0\right):={\mathcal {X}}$ and ${\mathcal {X}}_{\mu }:={\text {dom}}\left(A^{\mu }\right)$, equipped with the following norm

$$\begin{aligned} \left\| u\right\| _{{\mathcal {X}}_{\mu }}:=\sum _{j=0}^{\mu }{\left\| A^j u\right\| _{{\mathcal {X}}}}. \end{aligned}$$

For $\mu \in [0,\infty )$, we define ${\mathcal {X}}_{\mu }:=\left[ {\mathcal {X}}_{\lfloor \mu \rfloor }, {\mathcal {X}}_{\lfloor \mu \rfloor +1}\right] _{\mu -\lfloor \mu \rfloor }$ by interpolation.

We sometimes consider ${\text {dom}}(A)$ as a Banach space. It is to be understood carrying the graph norm, same as ${\mathcal {X}}_1$.

### Runge–Kutta approximation and discrete stage derivative

An *m*-stage Runge–Kutta method is given by its Butcher tableau, characterized by ${\mathcal {Q}}\in {\mathbb {R}}^{m\times m}$ and ${\mathbf {b}}$, ${\mathbf {c}}\in {\mathbb {R}}^{m}$. The Runge–Kutta approximation of the problem (2.1a–2.1c) starts at $u^k_0:=u_0$ and then computes for $n\ge 0$ the stage vector $U^k_n \in {\mathcal {X}}^m$ and the step approximation $u^k_{n+1}\in {\mathcal {X}}$ by solving

2.7a $$\begin{aligned} U^k_n&= {\mathbf {1}}\, u^k_n+ k ({\mathcal {Q}}\otimes A_{\star }) U^k_n + k {\mathcal {Q}}F(t_n + k \,{\mathbf {c}}), \end{aligned}$$

2.7b $$\begin{aligned} ({\mathcal {I}} \otimes B ) U^k_n&=\varXi \left( t_n + k \,{\mathbf {c}}\right) , \end{aligned}$$

2.7c $$\begin{aligned} u^k_{n+1}&= u^k_n + k ({\mathbf {b}}^\top \otimes A_{\star }) U^k_n + k {\mathbf {b}}^\top F(t_n + k \,{\mathbf {c}}). \end{aligned}$$

We have used the following notation (the spaces ${\mathcal {Y}}$ and ${\mathcal {Z}}$ are generic):

- For a function $G:[0,T]\rightarrow {\mathcal {Y}}$ we write
  $$\begin{aligned} G(t_n+k{\mathbf {c}}):= \left( \begin{array}{ccc} G(t_n+k c_1),&\ldots&, G(t_n+ k c_m)\end{array}\right) ^\top \in {\mathcal {Y}}^m. \end{aligned}$$
- For a matrix ${\mathcal {S}}\in {\mathbb {R}}^{m\times m}$ and an operator $C:{\mathcal {Y}}\rightarrow {\mathcal {Z}}$ we write
  $$\begin{aligned} {\mathcal {S}}\otimes C:= \left[ \begin{array}{ccc} {\mathcal {S}}_{11} C &{} \cdots &{} {\mathcal {S}}_{1m} C \\ \vdots &{} &{} \vdots \\ {\mathcal {S}}_{m1} C &{} \cdots &{} {\mathcal {S}}_{mm} C \end{array}\right] : {\mathcal {Y}}^m \rightarrow {\mathcal {Z}}^m. \end{aligned}$$
- For the vector ${\mathbf {b}}$ and an operator $C:{\mathcal {Y}} \rightarrow {\mathcal {Z}}$ we write
  $$\begin{aligned} {\mathbf {b}}^\top \otimes C := \left[ \begin{array}{ccc} b_1 C&\cdots&b_m C \end{array}\right] : {\mathcal {Y}}^m \rightarrow {\mathcal {Z}}. \end{aligned}$$
- ${\mathcal {I}}$ is the $m\times m$ identity matrix, and ${\mathbf {1}}=(1,\cdots ,1)^\top$.
- We admit shortened expressions such as
  $$\begin{aligned} {\mathcal {Q}}F(t_n+k{\mathbf {c}})&:=({\mathcal {Q}}\otimes I) F(t_n+k{\mathbf {c}}),\\ {\mathbf {1}}\, u&:= ({\mathbf {1}}\otimes I) u,\\ {\mathbf {b}}^\top F(t_n+k{\mathbf {c}})&:=({\mathbf {b}}^\top \otimes I) F(t_n+k{\mathbf {c}}). \end{aligned}$$

The following lemma involving inversion of matrices of operators associated to an operator can be proved by taking the Jordan canonical form of the matrix ${\mathcal {S}}$.

#### Lemma 2.4

If $A:{\text {dom}}(A)\subset {\mathcal {X}}\rightarrow {\mathcal {X}}$ is a linear operator on a Banach space ${\mathcal {X}}$ and ${\mathcal {S}}\in {\mathbb {C}}^{m\times m}$ satisfies $\sigma (A) \cap \sigma ({\mathcal {S}}) = \emptyset$, then

$$\begin{aligned} {\mathcal {I}}\otimes A - {\mathcal {S}}\otimes I : ({\text {dom}}(A))^m \rightarrow \mathcal {X}^m \end{aligned}$$

is invertible. Furthermore, there exists a constant $C_{\mathcal {S}}$, depending only on ${\mathcal {S}}$, such that

$$\begin{aligned} \Vert ({\mathcal {I}}\otimes A - {\mathcal {S}}\otimes I)^{-1}\Vert _{\mathcal {X}^m\rightarrow {\mathcal {X}}^m} \le C_{{\mathcal {S}}} \,\left[ 1+\max _{\mu \in \sigma ({\mathcal {S}})} \Vert (A-\mu \,I)^{-1}\Vert _{{\mathcal {X}}\rightarrow \mathcal {X}}\right] ^m. \end{aligned}$$

Under Assumption 2.I, the internal stage computation in the RK method can be decomposed in the following form:

2.8a $$\begin{aligned} Y^k_n:&= ({\mathcal {I}}\otimes {\mathscr {E}}) \varXi (t_n+k\,{\mathbf {c}}), \end{aligned}$$

2.8b $$\begin{aligned} Z^k_n-k({\mathcal {Q}}\otimes A)Z^k_n&={\mathbf {1}}u^k_n-Y^k_n+k {\mathcal {Q}}(Y^k_n+F(t_n+k\,{\mathbf {c}})), \end{aligned}$$

2.8c $$\begin{aligned} U^k_n:&=Y^k_n+Z^k_n. \end{aligned}$$

In (2.8b) we look for $Z^k_n\in (\mathrm {dom}(A))^m$.

The stability function of the Runge–Kutta method is the rational function $r(z):=1+z{\mathbf {b}}^\top (I-z{\mathcal {Q}})^{-1}{\mathbf {1}}$. We will not consider the full class of Runge–Kutta methods, but will restrict our considerations to those satisfying the following Assumptions:

#### Assumption 2.II

(i) The matrix ${\mathcal {Q}}$ is invertible.
(ii) The stability function *r* does not have poles in $\{ z\,:\, \mathrm {Re}\,z<0\}$, and $\left| r(it)\right| \le 1$ for all $t \in {\mathbb {R}}$ (i.e., the method is *A*-stable). Equivalently, $|r(z)|<1$ for all *z* with negative real part.

We note that Assumption 2.II (i) implies that the following limit exists

$$\begin{aligned} \lim _{z\rightarrow \infty } r(z)=1-{\mathbf {b}}^\top {\mathcal {Q}}^{-1}{\mathbf {1}}=:r(\infty ). \end{aligned}$$

Assumption 2.II (ii) implies that

$$\begin{aligned} \sigma ({\mathcal {Q}})\subset {\mathbb {C}}_+:=\{ z\in {\mathbb {C}}\,:\, \mathrm {Re}\,z>0\}, \end{aligned}$$

and that *r* is a rational function with poles only in ${\mathbb {C}}_+$ and bounded at infinity.

The computation of the internal stages in the numerical approximation (2.7a–2.7c) requires the inversion of

$$\begin{aligned} {\mathcal {I}}\otimes I-k ({\mathcal {Q}}\otimes A) =({\mathcal {Q}}\otimes I)({\mathcal {Q}}^{-1}\otimes I-{\mathcal {I}}\otimes (k\,A)), \end{aligned}$$

as can be seen from the equivalent form (2.8a–2.8c).

If *A* is the infinitesimal generator of a $C_0$-semigroup and $\omega$ and *M* are given by Proposition 2.1 and if we choose (recall that $\sigma ({\mathcal {Q}})\subset {\mathbb {C}}_+$)

2.9 $$\begin{aligned} k_0< \omega ^{-1} d_0, \quad d_0:=\min \{ \mathrm {Re}\,\lambda \,:\, \lambda \in \sigma ({\mathcal {Q}}^{-1})\}, \end{aligned}$$

then the RK method can be applied for any $0 < k\le k_0$. By Proposition 2.1 and Lemma 2.4, it follows that

2.10 $$\begin{aligned} \Vert ({\mathcal {I}}\otimes I-k ({\mathcal {Q}}\otimes A))^{-1}\Vert _{{\mathcal {X}}^m \rightarrow {\mathcal {X}}^m} \le C_{{\mathcal {Q}}} \frac{M}{d_0-k_0\omega }, \quad \forall k\le k_0. \end{aligned}$$

Using Definition 2.2, we can define $r(k\, A)$ for an RK method satisfying Assumption 2.II and $k\le k_0$ satisfying (2.9). We then define

2.11 $$\begin{aligned} \rho _k(T):=\sup _{0\le n k\le T} \left\| r(kA)^{n}\right\| _{{\mathcal {X}} \rightarrow {\mathcal {X}}}. \end{aligned}$$

This quantity is relevant for the study of the error propagation in the Runge–Kutta method.

Given an RK method, we consider the following matrix-valued rational function

2.12 $$\begin{aligned} \delta (z):= \left( {\mathcal {Q}}+ \frac{z}{1-z} {\mathbf {1}}{\mathbf {b}}^\top \right) ^{-1} = {\mathcal {Q}}^{-1}-\frac{z}{1-r(\infty )z}{\mathcal {Q}}^{-1}{\mathbf {1}}{\mathbf {b}}^\top {\mathcal {Q}}^{-1}. \end{aligned}$$

(The verification that these two formulas correspond to the same matrix is simple by using the Sherman–Morrison–Woodbury formula.) This matrix is related to the discrete differentiation process associated to an RK method satisfying Assumption 2.II: on the one hand $k^{-1}\delta (z)$ is the discrete symbol associated to the discrete operational calculus built with the RK method [19]; on the other hand, a direct interpretation of this symbol is possible using the *Z*-transformation (see [14, Sect. 6]). Given a sequence $U:=\{ U_n\}$ (tagged from $n\ge 0$) on a space, its *Z*-transform $\mathcal{Z} \{ U_n\}$ is the formal series

$$\begin{aligned} {\widehat{U}}(z):=\sum _{n=0}^\infty U_n z^n. \end{aligned}$$

For a detailed treatment on formal power series, see [12].

#### Definition 2.5

Let $U:=\{ U_n\}$ and $V:=\{ V_n\}$ be two sequences in ${\mathcal {X}}^m$ and let ${\widehat{U}}$ and ${\widehat{V}}$ be their respective *Z*-transforms. If

$$\begin{aligned} k^{-1} \delta (z){\widehat{U}}(z)={\widehat{V}}(z), \end{aligned}$$

we write

$$\begin{aligned} \partial ^k U=V, \quad U=(\partial ^k)^{-1}V. \end{aligned}$$

The above definition is consistent with the RK discrete operational calculus of Lubich and Ostermann, see Sect. 8.1 and [19]. We now show an explicit form of the computation of $\partial ^k$ and its inverse.

#### Lemma 2.6

If $U=\{U_n\}$ is a sequence in ${\mathcal {X}}^m$, then $X:=(\partial ^k)^{-1}U$ can be computed with the recurrence

2.13 $$\begin{aligned} x_0:=0, \quad \begin{array}{l} \;\,\; X_n:={\mathbf {1}}x_n+k{\mathcal {Q}}U_n,\\ x_{n+1}:=x_n+k{\mathbf {b}}^\top U_n =r(\infty ) x_n+{\mathbf {b}}^\top {\mathcal {Q}}^{-1} X_n, \end{array} \end{aligned}$$

and $V:=\partial ^k U$ can be computed with the inverse recurrence

2.14 $$\begin{aligned} u_0:=0, \quad \begin{array}{l} \;\;\,V_n:=k^{-1}{\mathcal {Q}}^{-1} (U_n-{\mathbf {1}}u_n),\\ u_{n+1}:=u_n+k{\mathbf {b}}^\top V_n=r(\infty ) u_n+{\mathbf {b}}^\top {\mathcal {Q}}^{-1}U_n. \end{array} \end{aligned}$$

#### Proof

The proof of (2.13) is a simple exercise in *Z*-transforms, while (2.14) follows from (2.13) by writing $U_n$ in terms of $X_n$ (and changing names to the sequences). $\square$

The first result of Lemma 2.6 expresses the fact that if we apply the RK method to the equation

$$\begin{aligned} \dot{x}(t)=u(t), \quad x(0)=0, \quad \text{ i.e.}, \quad x(t)=\int _0^t u(\tau )\mathrm d\tau , \end{aligned}$$

and $X:=\{ X_n\}$ is the sequence of vectors of internal stages, then $X=(\partial ^k)^{-1} U$, where $U_n:=u(t_n+k{\mathbf {c}})$.

Finally we note that we call a Runge–Kutta method stiffly accurate, if it satisfies ${\mathbf {b}}^\top {\mathcal {Q}}^{-1}=\mathbf{e}_m^\top :=(0,\dots ,0,1)$. Stiffly accurate methods satisfy [we use that ${\mathcal {Q}}{\mathbf {1}}={\mathbf {c}}$, see (5.1)]

2.15 $$\begin{aligned} c_m={\mathbf {b}}^\top {\mathcal {Q}}^{-1}{\mathbf {c}}={\mathbf {b}}^\top {\mathcal {Q}}^{-1}{\mathcal {Q}}{\mathbf {1}}= {\mathbf {b}}^\top {\mathbf {1}}=1, \end{aligned}$$

and $$r(\infty )=0.$$

For stiffly accurate methods, taking the discrete derivative of a stage vector consisting of samples taken from a continuous function is particularly simple:

#### Lemma 2.7

Let $t \mapsto F(t)$ be a continuous function with $F(0) = 0$. For stiffly accurate RK methods the sequence $G:=\partial ^k F$ with $F_n=F(t_n+k{\mathbf {c}})$ satisfies

$$\begin{aligned} G_n=k^{-1}{\mathcal {Q}}^{-1}(F(t_n+k{\mathbf {c}})-{\mathbf {1}}F(t_n)). \end{aligned}$$

#### Proof

For stiffly accurate methods we have $r(\infty )=0$ and therefore

$$\begin{aligned} \delta (z)={\mathcal {Q}}^{-1}-z {\mathcal {Q}}^{-1} {\mathbf {1}}{\mathbf {b}}^\top {\mathcal {Q}}^{-1} ={\mathcal {Q}}^{-1} -z {\mathcal {Q}}^{-1} {\mathbf {1}}{\mathbf {e}}_m^\top . \end{aligned}$$

However, since $c_m=1$, we have ${\mathbf {e}}_m^\top F(t_{n-1}+ k{\mathbf {c}})=F(t_{n-1}+k c_m)=F(t_n),$ which proves the result. $\square$

We also make the following optional assumption, which allows us to increase the convergence order in some cases.

#### Assumption 2.III

For all $t\in {\mathbb {R}}, t \ne 0$ the stability function satisfies $\left| r(it)\right| < 1$ and $r(\infty )<1$.

## Error estimates

We are now in a position to formulate the main results of this article and put them into context with previous results, most notably from [1].

To simplify notation, we will write for $v\in \mathcal C([0,T];{\mathcal {X}}_\mu )$ with $\mu \ge 0,$

$$\begin{aligned} \Vert v\Vert _{T,\mu }:=\max _{\tau \in [0,T]} \Vert v(\tau )\Vert _{{\mathcal {X}}_\mu }. \end{aligned}$$

For functions $f:[0,T]\rightarrow {\mathcal {Y}}$, we will write $(\partial ^{-1} f)(t):=\int _0^t f(\tau )\mathrm d\tau$, where ${\mathcal {Y}}$ denotes a generic Banach space.

### The estimates of Alonso-Mallo and Palencia

The following two propositions summarize the results of Alonso-Mallo and Palencia [1], rewritten with the notation of the present paper. The ‘proofs’ which we provide clarify how notation needs to be adapted and how the hypotheses of the main results of [1] are satisfied in our context.

#### Proposition 3.1

([1, Theorem 1]) Let Assumption 2.I hold and assume that the exact solution *u* satisfies $u \in {\mathcal {C}}^{p+1}\left( \left[ 0,T\right] ,{\mathcal {X}}_{\mu }\right)$ for some $\mu \ge 0$. Let $\{ u^k_n\}$ denote the Runge–Kutta approximation from (2.7a–2.7c). Then there exist constants $k_0 > 0$ and $C>0$ such that for $0<k\le k_0$ and $0< n k\le T$ the following estimate holds:

3.1 $$\begin{aligned} \Vert u(t_n) - u^k_n\Vert _{{\mathcal {X}}}&\le C T \rho _{k}(T) k^{\min \{q+\mu ,p\}} \Big (\sum _{\ell =q+1}^{p} \Vert u^{(\ell )}\Vert _{T,\mu } + \Vert u^{(p+1)}\Vert _{T,0}\Big ) . \end{aligned}$$

The constant *C* depends on the Runge–Kutta method, $\mu$, and the constants *M* and $\omega$ from (2.2). The constant $k_0$ depends only on $\omega$ and the Runge–Kutta method.

#### Proof

We only remark on the differences in notation. A different definition of interpolation spaces is given in [1], but the proof only relies on estimates of the form (2.6). The choice of $k_0$ follows from the fact that it is only needed to ensure that $(I - k\,{\mathcal {Q}}\otimes A)$ is invertible, see (2.10). The assumption $\mu \le p-q$ in [1, Theorem 1] can be replaced by using the rate $\min \{p,q+\mu \}$ in (3.1) as the spaces ${\mathcal {X}}_{\mu } \subseteq {\mathcal {X}}_{p-q}$ are nested for $\mu \ge p-q$. We also lowered the regularity requirements on the highest derivative compared to their stated result. The fact that this holds true follows from inspection of the proof. See also Lemma 5.9 for the key ingredient. $\square$

For certain Runge–Kutta methods, these estimates can be improved:

#### Proposition 3.2

([1, Theorem 2]) Let the assumptions of Proposition 3.1 hold and assume that, in addition, the RK method satisfies Assumption 2.III. Then there exist constants $k_0 > 0$, $C>0$ such that for $0<k\le k_0$ and $0< n k\le T$ the following improved estimate holds:

3.2 $$\begin{aligned} \Vert u(t_n) - u^k_n\Vert _{{\mathcal {X}}}&\le C (1+T) \rho _{k}(T) k^{\min \{ q+ \mu +1,p\}} \sum _{\ell =q+1}^{p+1} \Vert u^{(\ell )}\Vert _{T,\mu }. \end{aligned}$$

The constant *C* depends on the Runge–Kutta method, $\mu$, and the constants *M* and $\omega$ from (2.2); $k_0$ depends only on the constant $\omega$ and the Runge–Kutta method.

#### Proof

Again, this is just a reformulation of [1, Theorem 2]. We first note that, due to our assumption on $r(\infty )$, we are always in the case $m=0$ of [1]. Since we assumed that on the imaginary axis $\left| r(it)\right| <1$ for $0\ne t \in {\mathbb {R}}$, we directly note that for sufficiently small $k_0$, all the zeros of $r(z)-1$ except $z=0$ satisfy $\mathrm {Re}\, z > k_0 \omega$. By the resolvent bound (2.2) we can therefore estimate for $k\le k_0$

$$\begin{aligned} \left\| (z I - k A)^{-1}\right\| _{{\mathcal {X}} \rightarrow {\mathcal {X}}}\le \frac{M}{\mathrm {Re}\,z - k_0 \omega }, \quad \text{ if } \mathrm {Re}\,z \,\ge \, k_0 \omega , \end{aligned}$$

i.e., we have a uniform resolvent bound in the set $Z_{\alpha ,\delta }$ in [1]. We also note that we reformulated the convergence rate such that we do not have the restriction $\mu \le p-q-1$, since the exceptional cases are already covered by Proposition 3.1. $\square$

#### Remark 3.3

The assumption $\left| r(z)\right| <1$ for ${\text {Re}}(z) \le 0$ and $r(\infty )\ne 1$ is satisfied by the Radau IIA family of Runge–Kutta methods, but is violated by the Gauss methods, which satisfy $\left| r(z)\right| =1$ on the imaginary axis.

### New results in this article

In this section we present some a priori estimates for the convergence of Runge–Kutta methods when applied to the abstract problem (2.1a–2.1c). These can be seen as a continuation of [1] to the case where the boundary conditions are not given exactly but stem from computing discrete integrals and differentials using the same Runge–Kutta method.

#### Theorem 3.4

(Integrated estimate) Let *u* solve (2.1a–2.1c) with $u_0=0$ and assume that for some $\mu \ge 0$ we have

$$\begin{aligned} u&\in {\mathcal {C}}^{p}([0,T];{\mathcal {X}}_\mu ), \quad {\mathscr {E}}\varXi , F \in {\mathcal {C}}^{p-1}([0,T];{\mathcal {X}}_\mu ) \cap {\mathcal C}^{p}([0,T];{\mathcal {X}}_0). \end{aligned}$$

Set $x:=\partial ^{-1} u$. Let $U^k=\{ U^k_n\}$ and let $u^k=\{ u^k_n\}$ be the discrete approximation given by (2.7a–2.7c) for a method satisfying Assumption 2.II. If $X^k:=(\partial ^k)^{-1} U^k$ and we define $x^k=\{x^k_n\}$ with the recurrence

$$\begin{aligned} x_0^k:=0, \quad x_{n+1}^k:=r(\infty ) x_n^k + {\mathbf {b}}^\top {\mathcal {Q}}^{-1} X_n^k, \end{aligned}$$

then there exists a constant $k_0>0$ such that for all $k<k_0$ and $n \in {\mathbb {N}}$ with $nk\le T$ the following estimate holds:

$$\begin{aligned} \Vert x(t_n)& -x^k_n\Vert _{{\mathcal {X}}} \le \\ &C T \rho _k(T) k^{\min \{q+\mu +1,p\}} \; \Bigg [\sum _{\ell =q}^{p-1} \left( \Vert u^{(\ell )}\Vert _{T,\mu } + \Vert {\mathscr {E}}\varXi ^{(\ell )}\Vert _{T,\mu } + \Vert F^{(\ell )}\Vert _{T,\mu }\right) \\ & \quad \quad \quad \quad \quad \quad \quad \quad \; \; +\left( \Vert u^{(p)}\Vert _{T,\mu } + \Vert {\mathscr {E}}\varXi ^{(p)}\Vert _{T,0} + \Vert F^{(p)}\Vert _{T,0}\right) \Bigg ] \\ &+ C \,T^2 \rho _k(T) k^{p}\left( \Vert {\mathscr {E}}\varXi ^{(p)}\Vert _{T,0} + \Vert F^{(p)}\Vert _{T,0} \right) . \end{aligned}$$

If Assumption 2.III holds and if we assume the stronger regularities

$$\begin{aligned} u\in {\mathcal {C}}^{p+1}([0,T];{\mathcal {X}}_\mu ), \quad F \in \mathcal {C}^{p}([0,T];{\mathcal {X}}_\mu ), \quad {\mathscr {E}}\varXi \in \mathcal {C}^{p}([0,T];{\mathcal {X}}_\mu ), \end{aligned}$$

then

$$\begin{aligned}&\Vert x(t_n)-x^k_n\Vert _{{\mathcal {X}}} \le \\&\quad C (1+T) \rho _k(T) k^{\min \{q+\mu +2,p\}} \Bigg [\sum _{\ell =q}^{p} \Vert u^{(\ell )}\Vert _{T,\mu }+\Vert {\mathscr {E}}\varXi ^{(\ell )}\Vert _{T,\mu } + \Vert F^{(\ell )}\Vert _{T,\mu } + \Vert u^{(p+1)}\Vert _{T,\mu } \Bigg ] \\&\quad + C \,T^2 \rho _k(T) k^{p}\left( \Vert {\mathscr {E}}\varXi ^{(p)}\Vert _{T,0} \quad + \Vert F^{(p)}\Vert _{T,0} \right) . \end{aligned}$$

The constant $k_0$ depends only on $\omega$ from (2.2) and the Runge–Kutta method. If $\omega =0$ then $k_0$ can be chosen arbitrarily large. *C* depends on $\omega$, *M* from (2.2), the Runge–Kutta method, and $\mu$.

#### Theorem 3.5

(Differentiated estimate) Let *u* solve (2.1a–2.1c) with $u_0=0$ and assume ${\dot{u}}(0)=0$. Assume that for some $\mu \ge 0$ we have

$$\begin{aligned} u\in {\mathcal {C}}^{p+1}([0,T];{\mathcal {X}}_\mu )\cap {\mathcal {C}}^{p+2}([0,T];{\mathcal {X}}_0) , \quad {\mathscr {E}}\varXi , F \in \mathcal {C}^{p}([0,T];{\mathcal {X}}_\mu ) \cap {{\mathcal {C}}}^{p+1}([0,T];\mathcal {X}_0), \end{aligned}$$

and let $v:=\dot{u}$. Let $U^k=\{ U^k_n\}$ and $u^k=\{ u^k_n\}$ be the discrete approximation given by (2.7a–2.7c) for a stiffly accurate method satisfying Assumption 2.II.

If $V^k:=\partial ^kU^k$ and $v^k_n={\mathbf {e}}_m^\top V^k_{n-1}$, then there exists a constant $k_0>0$ such that for all $k<k_0$ and $n\ge 1$ such that $nk\le T$ the following estimate holds:

$$\begin{aligned} \Vert v(t_n)-v^k_n\Vert _{{\mathcal {X}}} + \Vert A_\star (u(t_n)-u^k_n)\Vert _{{\mathcal {X}}} \le C T \rho _k(T) k^{\min \{q+\mu ,p\}-1} &\Bigg (\!\sum _{\ell =q+1}^{p} \big ( \Vert u^{(\ell +1)}\Vert _{T,\mu }+\Vert {\mathscr {E}}\varXi ^{(\ell )}\Vert _{T,\mu } +\Vert F^{(\ell )}\Vert _{T,\mu }\bigg ) \\& + \Vert u^{(p+2)}\Vert _{T,0}+\Vert {\mathscr {E}}\varXi ^{(p+1)}\Vert _{T,0} +\Vert F^{(p+1)}\Vert _{T,0} \Bigg ). \end{aligned}$$

If, in addition, the method satisfies Assumption 2.III and

$$\begin{aligned} u\in {\mathcal {C}}^{p+2}([0,T];{\mathcal {X}}_\mu ) , \quad {\mathscr {E}}\varXi , F \in {\mathcal {C}}^{p+1}([0,T];{\mathcal {X}}_\mu ) \cap {\mathcal C}^{p+2}([0,T];{\mathcal {X}}_0), \end{aligned}$$

then

$$\begin{aligned} & \Vert v(t_n)-v^k_n\Vert _{{\mathcal {X}}}+ \Vert A_\star (u(t_n)-u^k_n)\Vert _{{\mathcal {X}}} \\&\quad \le C (1+T) \rho _k(T) k^{\min \{q+\mu ,p\}} \Big (\sum _{\ell =q+1}^{p+1} \big ( \Vert u^{(\ell+1)}\Vert _{T,\mu }+\Vert {\mathscr {E}}\varXi ^{(\ell )}\Vert _{T,\mu } +\Vert F^{(\ell )}\Vert _{T,\mu }\big ) \\&\qquad \qquad \qquad \qquad \qquad \qquad \;+\Vert {\mathscr {E}}\varXi ^{(p+2)}\Vert _{T,0} +\Vert F^{(p+2)}\Vert _{T,0} \Big ). \end{aligned}$$

The constant $k_0$ depends only on $\omega$ from (2.2) and the Runge–Kutta method. If $\omega =0$, then $k_0$ can be chosen arbitrarily large. *C* depends on $\omega$, *M* from (2.2), the Runge–Kutta method, and $\mu$.

#### Remark 3.6

Most of the effort in proving the above theorem is done in order to obtain a convergence rate higher than *q*, even though the constraint in the stages is approximated only with order *q*. This is possible by exploiting the additional structure of the discretization error of the side constraint.

#### Remark 3.7

We formulated all our results for homogeneous initial conditions, since it is sufficient for our purposes in time domain BEM and convolution quadrature. It should be possible to generalize these results to the case of $u_0 \in {\text {dom}}(A^{s})$ for sufficiently large $s \ge 1$ by considering the evolution of the semigroup with inhomogeneous side constraint but homogeneous initial condition and the semigroup of homogeneous constraint but inhomogeneous $u_0$ separately.

#### Remark 3.8

The loss of order by 1 in Theorem 3.5 compared to Propositions 3.1 and 3.2 is to be expected. Indeed, if we look at the case $u \in {\text {dom}}(A^{\mu })$ for $\mu \ge 1$, this means $A_{\star }u \in {\text {dom}}(A^{\mu -1})$. Applying Proposition 3.2 to this semigroup then also gives a reduced order of $k^{\min (q+\mu ,p)}$.

## Some computations related to the main theorems

We will collect the sampled data and the stage and step parts of the solutions in four formal series

4.1a $$\begin{aligned} {\widehat{F}}^k(z)&:=\sum _{n=0}^\infty F(t_n+k{\mathbf {c}})z^n,\quad{\widehat{\varXi }}^k(z):=\sum _{n=0}^\infty \varXi (t_n+k{\mathbf {c}})z^n, \end{aligned}$$

4.1b $$\begin{aligned} {\widehat{U}}^k(z)&:=\sum _{n=0}^\infty U^k_n z^n, \quad {\widehat{u}}^k(z):=\sum _{n=0}^\infty u^k_n z^n. \end{aligned}$$

If the data functions are polynomially bounded in time, the series in (4.1a) are convergent (in ${\mathcal {X}}^m$ and $\mathcal M^m$ respectively) with at least unit radius of convergence. Because of the equivalent formulation of the numerical method in the form (2.8a–2.8c), and using (2.10), it follows that for $k\le k_0$ [with $k_0$ chosen using (2.9)], the numerical solution is at least bounded in the form $\left\| U^k_n\right\| _{{\mathcal {X}}} \lesssim C^{n}$. Thus, the two series in (4.1b) also converge on a sufficiently small disk.

### Proposition 4.1

The sequences $\{ U^k_n\}$ and $\{u^k_n\}$ satisfy equations (2.7a–2.7c) if and only if

4.2a $$\begin{aligned} k^{-1} \delta (z) {\widehat{U}}^k(z)&=(\mathcal I\otimes A_\star ) {\widehat{U}}^k(z)+ {\widehat{F}}^k(z) +\frac{k^{-1}}{1-r(\infty ) z} {\mathcal {Q}}^{-1}{\mathbf {1}}u_0, \end{aligned}$$

4.2b $$\begin{aligned} ({\mathcal {I}}\otimes B){\widehat{U}}^k(z)&={\widehat{\varXi }}^k(z), \end{aligned}$$

4.2c $$\begin{aligned} {\widehat{u}}^k(z)&=\frac{z}{1-r(\infty ) z} {\mathbf {b}}^\top {\mathcal {Q}}^{-1} {\widehat{U}}^k(z)+\frac{1}{1-r(\infty )z}u_0^k. \end{aligned}$$

### Proof

Let us start by proving a simple result: *the discrete equations* (2.7a) *and* (2.7c) *hold if and only if* (2.7a) *and*

4.3 $$\begin{aligned} u^k_{n+1}=r(\infty ) u^k_n+{\mathbf {b}}^\top {\mathcal {Q}}^{-1} U^k_n \end{aligned}$$

*hold.* To see this, note that (2.7a) is equivalent to

$$\begin{aligned} {\mathcal {Q}}^{-1}(U^k_n-{\mathbf {1}}u^k_n) =k(({\mathcal {I}}\otimes A_\star )U^k_n+F(t_n+k{\mathbf {c}})) \end{aligned}$$

and therefore (2.7a) and (2.7c) imply

$$\begin{aligned} {\mathbf {b}}^\top {\mathcal {Q}}^{-1} (U^k_n-{\mathbf {1}}u^k_n) =k(({\mathbf {b}}^\top \otimes A_\star )U^k_n+{\mathbf {b}}^\top F(t_n+k{\mathbf {c}})) =u^k_{n+1}-u^k_n, \end{aligned}$$

or equivalently (4.3). The reciprocal statement is proved similarly. The recurrence (4.3) is equivalent to (4.2c). At the same time, the recurrence (2.7a) is equivalent to

4.4 $$\begin{aligned} k^{-1}{\mathcal {Q}}^{-1} ({\widehat{U}}^k(z)-{\mathbf {1}}{\widehat{u}}^k(z)) =(\mathcal {I}\otimes A_\star ){\widehat{U}}^k(z)+{\widehat{F}}^k(z). \end{aligned}$$

After inserting (4.2c) into (4.4), the formula (4.2a) follows. $\square$

Proposition 4.1 is a rephrasing of [28, Lemma 3.19], where the computation is also laid out in more detail. Note how equations (4.2a)–(4.2b) relate strongly to (2.1a–2.1c), with the discrete symbol $k^{-1}\delta (z)$ playing the role of the time derivative and

$$\begin{aligned} \frac{k^{-1}}{1-r(\infty )z} {\mathcal {Q}}^{-1}{\mathbf {1}}\end{aligned}$$

playing the role of a discrete Dirac delta at time $t=0$.

### Lemma 4.2

([2, Lemma 2.6]) If the matrix ${\mathcal {Q}}$ of the RK method is invertible, then for $\left| z\right| < 1$

$$\begin{aligned} \sigma (\delta (z)) \subseteq \sigma ({\mathcal {Q}}^{-1}) \cup \{ w \in {\mathbb {C}}: r(w) z = 1 \}. \end{aligned}$$

In particular, if the Runge–Kutta method is A-stable (Assumption 2.II), then $\sigma (\delta (z))\subset {\mathbb {C}}_+$.

We need a corollary to the previous result:

### Corollary 4.3

Let Assumption 2.II hold. Then, for all $r_0<1$, there exists a constant $d>0$ such that for all $\left| z\right| <r_0$ there holds

$$\begin{aligned} \sigma \big (\delta (z)\big ) \subset \Big \{ w \in {\mathbb {C}}_+: {\text {Re}}(w) > d \Big \}. \end{aligned}$$

### Proof

In view of of Lemma 4.2, since $\sigma ({\mathcal {Q}})$ is finite, independent of *z*, and contained in ${\mathbb {C}}_+$, we are mainly concerned with the set $\{ w \in {\mathbb {C}}: r(w) z = 1 \}$. We first note that

$$\begin{aligned} \bigcup _{\left| z\right| \le r_0} \{ w \in {\mathbb {C}}: r(w) z = 1 \} \subseteq \{ w \in {\mathbb {C}}: \left| r(w)\right| \ge 1/r_0 \}. \end{aligned}$$

Second, we observe that by taking $d_0$ small enough, we can ensure that $w \mapsto r(w)$ is continuous for ${\text {Re}}(w)\le d_0$ and thus

$$\begin{aligned} \{ w \in {\mathbb {C}}: \left| r(w)\right| \ge 1/r_0 \} \cap \{w \in {\mathbb {C}}: {\text {Re}}(w)\le d_0\}&= {r|_{\{{\text {Re}}(w)\le d_0\}}}^{-1}\big ([1/r_0,\infty )\big ) \end{aligned}$$

is a closed set. Third, by considering the limit along the imaginary axis, we get

$$\begin{aligned} \left| r(\infty )\right| =\lim _{n \rightarrow \infty } \left| r(in)\right| \le 1. \end{aligned}$$

Thus, for $\left| w\right|$ sufficiently large, it holds that $\left| r(w)\right| \le 1/r_0$.

Overall, we get that

$$\begin{aligned} \{ w \in {\mathbb {C}}: \left| r(w)\right| \ge 1/r_0 \} \cap \{w \in {\mathbb {C}}: {\text {Re}}(w)\le d_0\} \end{aligned}$$

is a compact set with empty intersection with the imaginary axis. Thus, it must have a positive distance from it. These observations and Lemma 4.2 conclude the proof. $\square$

### Lemma 4.4

Let Assumptions 2.I and 2.II hold. For $r_0<1$, there exists $k_0=k_0(\omega ,r_0)>0$ such that for all $k\le k_0$ and $|z|\le r_0$ the problem

4.5a $$\begin{aligned} -k^{-1}\delta (z) {\widehat{U}} + ({\mathcal {I}}\otimes A_\star ) {\widehat{U}}&={\widehat{F}}, \end{aligned}$$

4.5b $$\begin{aligned} ({\mathcal {I}}\otimes B) {\widehat{U}}&={\widehat{\varXi }} \end{aligned}$$

has a unique solution for arbitrary ${\widehat{F}}\in {\mathcal {X}}^m$ and ${\widehat{\varXi }}\in {\mathcal {M}}^m$. If $\omega =0$ in Proposition 2.1, then there are no restrictions on *k*, and the results holds for all $|z|<1$.

### Proof

Assume first that ${\mathcal {S}}\in {\mathbb {C}}^{m\times m}$ is such that $\sigma ({\mathcal {S}}) \subset \{ z\,:\, \mathrm {Re}\,z>\omega \}$ and consider the problem

4.6a $$\begin{aligned} -({\mathcal {S}} \otimes I) {\widehat{U}} + ({\mathcal {I}}\otimes A_\star ) {\widehat{U}}&={\widehat{F}}, \end{aligned}$$

4.6b $$\begin{aligned} ({\mathcal {I}}\otimes B) {\widehat{U}}&={\widehat{\varXi }}. \end{aligned}$$

Take first ${\widehat{V}}:=({\mathcal {I}}\otimes {\mathscr {E}}){\widehat{\varXi }}$ (where ${\mathscr {E}}$ is the lifting operator of Assumption 2.I) and then seek ${\widehat{W}}\in (\mathrm {dom}\,(A))^m$ satisfying

$$\begin{aligned} -({\mathcal {S}}\otimes I){\widehat{W}}+({\mathcal {I}}\otimes A){\widehat{W}} ={\widehat{F}}+(({\mathcal {S}}-{\mathcal {I}})\otimes I){\widehat{V}}. \end{aligned}$$

This problem is uniquely solvable by Lemma 2.4, since $\sigma (A)\subset \{ z\,:\,\mathrm {Re}\,z\le \omega \}$ and therefore $\sigma (A)\cap \sigma ({\mathcal {S}})=\emptyset .$ We then define ${\widehat{U}}:={\widehat{V}}+{\widehat{W}}$, which solves (4.6a, 4.6b). To see uniqueness, one observes that the difference of two solutions of (4.6a, 4.6b) solves the homogeneous problem ($\widehat{\varXi }=0$ and $\widehat{F}=0$) for which uniqueness was established in Lemma 2.4.

By Corollary 4.3, the union of the spectra of $\delta (z)$ for $|z|\le r_0$ has a positive distance $d(r_0)>0$ from the imaginary axis. If we take $k_0< d(r_0)/\omega$, then $\sigma (k^{-1}\delta (z))\subset \{ s\,:\, \mathrm {Re}\,s>\omega \}$ for all $|z|\le r_0$ and $k\le k_0$. When $\omega =0$, we can take any $k_0$. By the previous considerations this implies unique solvability. $\square$

### Proposition 4.5

Let $U^k=\{U^k_n\}$ and $u^k=\{u^k_n\}$ be sequences satisfying (2.7a–2.7c) with $u^k_0=0$. The sequence $V^k=\{ V^k_n\}=\partial ^k U^k$ satisfies

4.7a $$\begin{aligned} V^k_n&= {\mathbf {1}}\, v^k_n+ k ({\mathcal {Q}}\otimes A_{\star }) V^k_n + k {\mathcal {Q}}G_n^k, \end{aligned}$$

4.7b $$\begin{aligned} ({\mathcal {I}}\otimes B ) V^k_n&=\varTheta _n^k, \end{aligned}$$

4.7c $$\begin{aligned} v^k_{n+1}&= r(\infty ) v^k_n + {\mathbf {b}}^\top {\mathcal {Q}}^{-1} V^k_n, \end{aligned}$$

for data $v^k_0=0$, $G^k=\{G^k_n\}:=\partial ^k \{ F(t_n+k{\mathbf {c}})\}$, and $\varTheta ^k=\{ \varTheta ^k_n\}:=\partial ^k \{ \varXi (t_n+k{\mathbf {c}})\}$. Moreover,

4.8a $$\begin{aligned} ({\mathcal {I}}\otimes A_\star ) U^k_n&=V^k_n-F(t_n+k{\mathbf {c}}), \end{aligned}$$

4.8b $$\begin{aligned} V^k_n&=k^{-1}{\mathcal {Q}}^{-1}(U^k_n-{\mathbf {1}}u^k_n). \end{aligned}$$

### Proof

Recall that Eqs. (2.7a) and (2.7c) are equivalent to (2.7a) and (4.3), as shown in the proof of Proposition 4.1. Moreover, the latter equations are equivalent to (4.2a–4.2c) in the *Z*-domain. In the present case we have $u_0=0$. For a given square matrix ${\mathcal {P}}\in {\mathbb {C}}^{m\times m}$ and an operator *C*, we have

$$\begin{aligned} ({\mathcal {P}}\otimes I)({\mathcal {I}}\otimes C)={\mathcal {P}}\otimes C=({\mathcal {I}}\otimes C)({\mathcal {P}}\otimes I), \end{aligned}$$

which proves that

4.9a $$\begin{aligned} k^{-1} \delta (z) {\widehat{V}}^k(z)&=({\mathcal {I}}\otimes A_\star ) {\widehat{V}}^k(z)+ {\widehat{G}}^k(z), \end{aligned}$$

4.9b $$\begin{aligned} ({\mathcal {I}}\otimes B){\widehat{V}}^k(z)&={\widehat{\varTheta }}^k(z), \end{aligned}$$

4.9c $$\begin{aligned} {\widehat{v}}^k(z)&=\frac{z}{1-r(\infty ) z} {\mathbf {b}}^\top {\mathcal {Q}}^{-1} {\widehat{V}}^k(z). \end{aligned}$$

By Proposition 4.1, Eq. (4.9a–4.9c) are equivalent to (4.7a–4.7c). Finally (4.8a) follows from (4.2a), while (4.8b) follows from (4.4) and (4.8a). $\square$

### Proposition 4.6

Let $U^k=\{U^k_n\}$ and $u^k=\{u^k_n\}$ be sequences satisfying (2.7a–2.7c) with $u^k_0=0$. The sequence $X^k=\{X^k_n\}=(\partial ^k)^{-1} U^k$ satisfies

4.10a $$\begin{aligned} X^k_n&= {\mathbf {1}}\, x^k_n+ k ({\mathcal {Q}}\otimes A_{\star }) X^k_n + k {\mathcal {Q}}H_n^k, \end{aligned}$$

4.10b $$\begin{aligned} ({\mathcal {I}}\otimes B ) X^k_n&=\varGamma _n^k, \end{aligned}$$

4.10c $$\begin{aligned} x^k_{n+1}&= r(\infty ) x^k_n + {\mathbf {b}}^\top {\mathcal {Q}}^{-1} X^k_n \end{aligned}$$

4.10d $$\begin{aligned}&= x^k_n+k({\mathbf {b}}^\top \otimes A_\star )X^k_n+k {\mathbf {b}}^\top H_n^k, \end{aligned}$$

for data $$x^k_0:=0$$, $$H^k=\{H^k_n\}:=(\partial ^k)^{-1} \{ F(t_n+k{\mathbf {c}})\}$$, $$\varGamma ^k=\{\varGamma ^k_n\}:=(\partial ^k)^{-1} \{ \varXi (t_n+k{\mathbf {c}})\}$$.

### Proof

Follow the proof of Proposition 4.5. $\square$

## Some Lemmas regarding Runge–Kutta methods

In order to shorten the statements of the results of this section, in all of them we will understand that:

1. We have an RK method with coefficients ${\mathcal {Q}}, {\mathbf {b}}, {\mathbf {c}}$ satisfying Assumption 2.II (invertibility of ${\mathcal {Q}}$ and A-stability). The method has classical order *p* and stage order *q*.
2. We have an operator *A* in ${\mathcal {X}}$ that is the generator of a $C_0$-semigroup, characterized by the quantities *M* and $\omega$ of Proposition 2.1. The associated Sobolev tower $\{ {\mathcal {X}}_\mu \}$, obtained by interpolation of ${\text {dom}}(A^\mu)$ for positive integer values of $\mu$, will also be used.

The following lemma will be used at a key point in the arguments below.

### Lemma 5.1

Let *A* be a linear operator in ${\mathcal {X}}$ and *q* be a rational function bounded at infinity whose poles are outside $\sigma (A)$. The following properties hold:

- The operator *q*(*A*) maps ${\text {dom}} \left(A^\ell \right)$ to ${\text {dom}} \left( A^\ell \right)$ for all $\ell .$
- If $0\notin \sigma (A)$ and we define $p(z):=z^{-\ell } q(z)$, then $q(A)=p(A)A^\ell$ in ${\text {dom}} \left ( A^\ell \right )$.

### Proof

To prove (a), show first by induction on $\ell$ that $(A-\lambda I)^{-1}$ maps ${\text {dom}} \left( A^\ell \right)$ into ${\text {dom}}\left( A^{\ell +1} \right)$. Using this result for each of the factors in the definition (2.3) the result follows. To prove (b) note first that *p* is rational, bounded at infinity, and that $\sigma (A)$ does not intersect the set of poles of *p*. Using Definition 2.2, we have $p(A)=A^{-\ell } q(A)= q(A) A^{-\ell }$, and the result follows. $\square$

We start by recalling some simple facts about RK methods that we will need in the sequel. Using the notation ${\mathbf {c}}^{\ell }:=(c_{1}^{\ell },\ldots , c_{m}^{\ell })^\top$, the following equalities (order conditions) hold (see e.g. [1, 25]):

5.1a $$\begin{aligned} {\mathbf {c}}^{\ell }& =\ell {\mathcal {Q}}{\mathbf {c}}^{\ell -1}, \quad 0 \le 1\le \ell \le q, \end{aligned}$$

5.1b $$\begin{aligned} {\mathbf {b}}^\top {\mathcal {Q}}^{j} {\mathbf {c}}^{\ell }&=\frac{\ell !}{(j+\ell +1)!}, \quad 0 \le j+\ell \le p-1. \end{aligned}$$

Therefore,

5.2a $$\begin{aligned} \begin{aligned} {\mathbf {b}}^\top {\mathcal {Q}}^j({\mathbf {c}}^\ell -\ell {\mathcal {Q}}{\mathbf {c}}^{\ell -1}) = 0, \quad 0\le j\le p-\ell -1, \quad 1\le \ell \le p \end{aligned} \end{aligned}$$

5.2b $$\begin{aligned}\begin{aligned} \ell {\mathbf {b}}^\top {\mathbf {c}}^{\ell -1} =1, \quad 1 \le \ell \le p -1 . \end{aligned} \end{aligned}$$

For a stiffly accurate method we have (2.15) and therefore

5.3 $$\begin{aligned} {\mathbf {b}}^\top {\mathcal {Q}}^{-1}{\mathbf {c}}^\ell =c_m^\ell =1 \quad \forall \ell \in {\mathbb {N}}_0. \end{aligned}$$

The following result is well-known. We just summarize it for ease of reference later on.

### Lemma 5.2

(Discrete antiderivative and RK quadrature) Let $f:[0,T]\rightarrow {\mathcal {X}}$, $g:=\partial ^{-1}f$, $G^k=\{ G^k_n\}=(\partial ^k)^{-1} \{ f(t_n+k{\mathbf {c}})\}$ and $\{ g^k_n\}$ be given by the recursion

$$\begin{aligned} g^k_0:=0, \quad g_{n+1}^k:=g_n^k+k{\mathbf {b}}^\top f(t_n+k{\mathbf {c}}). \end{aligned}$$

For the errors $d^k_n:=g(t_n)-g^k_n$, and for *n* such that $nk\le T$, we have the estimates

5.4a $$\begin{aligned} \Vert d^k_n\Vert _{{\mathcal {X}}}&\le C T k^p \Vert f^{(p)}\Vert _{T,0}, \end{aligned}$$

5.4b $$\begin{aligned} \Vert d^k_n-d^k_{n-1}\Vert _{{\mathcal {X}}}&\le C k^{p+1} \max _{t_{n-1}\le t \le t_n}\Vert f^{(p)}(t)\Vert _{{\mathcal {X}}}. \end{aligned}$$

Additionally, at the stage level we have

5.4c $$\begin{aligned} \Vert k{\mathbf {b}}^\top g(t_n+k{\mathbf {c}})-k{\mathbf {b}}^\top G^k_n\Vert _{{\mathcal {X}}} \le C k^{p+1} \big (\Vert f^{(p-1)}\Vert _{T,0}+T \Vert f^{(p)}\Vert _{T,0}\big ). \end{aligned}$$

### Proof

Follows from the fact that the Runge–Kutta method defines a quadrature formula of order *p*. $\square$

### Estimates on rational functions of the operator

The following results in this section are adaptations from [1]. While they focus on the case $\beta =0$, we present the necessary generalizations to $\beta =\pm 1$. We will use the rational functions

5.5 $$\begin{aligned} r_{\ell ,\beta }(z)&:= z {\mathbf {b}}^\top (I-z{\mathcal {Q}})^{-1}{\mathcal {Q}}^\beta ({\mathbf {c}}^\ell -\ell {\mathcal {Q}}{\mathbf {c}}^{\ell -1}), \qquad \beta \in \{-1,0,1\}, \end{aligned}$$

5.6 $$\begin{aligned} s_n(z)&:=\sum _{j=0}^n r(z)^j. \end{aligned}$$

Note that these rational functions are bounded at infinity and that $r_{\ell ,\beta }(0)=0$. We will also use the vector-valued rational function

5.7 $$\begin{aligned} {\mathbf {g}}(z)^\top :=z {\mathbf {b}}^\top (I- z {\mathcal {Q}})^{-1}, \end{aligned}$$

and note that ${\mathbf {g}}(0)={\mathbf {0}}$ and $r(z)=1+\mathbf{g}(z)^\top {\mathbf {1}}$.

#### Lemma 5.3

The rational functions (5.5) satisfy

5.8 $$\begin{aligned} r_{\ell ,\beta }(z)=\mathcal {O}(|z|^{p+1-\ell -\beta }) \quad \text{ as } |z|\rightarrow 0, \qquad \ell \le p, \quad \beta \in \{0,1\}. \end{aligned}$$

The estimate (5.8) is also valid for $\beta =-1$ if the method is stiffly accurate.

#### Proof

For the case $\beta =0$, the proof is given in [1, Lemma 5]. For the case $\beta =\pm 1$, an analogous proof can be brought to fruition. Namely, one can expand the inverse in the definition of $r_{\ell ,\beta }$ into the Neumann series and apply the order conditions (5.2a, 5.2b). For $\beta =-1$, one has to use (5.3) for the leading term. $\square$

#### Lemma 5.4

If the RK method satisfies Assumption 2.III, then there exists a constant $k_0>0$ depending on the RK method and on $\omega$ such that for $\beta \in \{0,1\}$, $\ell \le p-\beta$, and all $0<k\le k_0$ and all *n* with $0\le n\,k \le T$, we have the estimate

5.9 $$\begin{aligned} \left\| s_n(k A) r_{\ell ,\beta }(k\,A)\right\| _{{\mathcal {X}}_\mu \rightarrow \mathcal {X}} \le C \,\rho _k(T) k^{\min \{\mu ,p-\ell -\beta \}} \end{aligned}$$

with $\rho _k(T)$ defined in (2.11). If $\ell =p$ and $\beta =1$, the left-hand side of (5.9) is bounded by $C\rho _k(T)$. The constant $C>0$ in (5.9) depends only on the Runge–Kutta method, *M* and $\omega$, $k_0$, $\ell$, and $\mu$, but is independent of *n* and *k*. If the Runge–Kutta method is stiffly accurate, then the estimate (5.9) also holds for $\beta =-1$. If $\omega =0$, then $k_0$ can be chosen arbitrarily.

#### Proof

We adapt the proof of [1, Lemma 6], which only covers the case $\beta =0$. Consider first the case $p-\ell -\beta \ge 0$ and take any integer $\mu$ such that $0\le \mu \le p-\ell -\beta$. Then

$$\begin{aligned} r_{\ell ,\beta }(z) \sum _{j=0}^n r(z)^j =(r(z)^{n+1}-1) q_{\ell ,\beta ,\mu }(z) z^\mu , \quad q_{\ell ,\beta ,\mu }(z):=\frac{r_{\ell ,\beta }(z)}{(r(z)-1) z^\mu }. \end{aligned}$$

By Lemma 5.3, the rational function $r_{\ell ,\beta }$ has a zero of order $p-\ell -\beta +1$ at $z=0$. The rational function $(r(z)-1)z^\mu$ has a zero of order $\mu +1\le p-\ell -\beta +1$ at $z=0$, and all other zeros are in ${\mathbb {C}}_+$ by A-stability and Assumption 2.III. This implies that the rational function $q_{\ell ,\beta ,\mu }$ has its poles in

5.10 $$\begin{aligned} \varLambda :=\{ z\ne 0\,:\, r(z)=1\}\cup \sigma ({\mathcal {Q}}^{-1})\subset {\mathbb {C}}_+. \end{aligned}$$

Therefore, for $k_0>0$ sufficiently small we get using (2.4):

5.11 $$\begin{aligned} \Vert q_{\ell ,\beta ,\mu }(kA)\Vert _{{\mathcal {X}} \rightarrow {\mathcal {X}}} \le C \qquad \forall 0 < k\le k_0, \end{aligned}$$

where *C* depends on *M*, $\omega$, $k_0$, and the RK method. By Lemma 5.1 we have

$$\begin{aligned} r_{\ell ,\beta }(kA)\!\! \sum _{j=0}^n r(kA)^jx=k^\mu (r(kA)^{n+1}-I) q_{\ell ,\beta ,\mu }(kA) A^\mu x \quad \forall x\in {\text {dom}} \left( A^\mu \right), \ k\le k_0. \end{aligned}$$

This, (5.11), and applying (2.11) to control $r(kA)^{n+1}$ by $\rho _k(T)$, proves (5.9) for integer $\mu \le p-\ell -\beta$. For larger integer values of $\mu$, the result does not need to be proved as the maximum rate is already attained. We just have to estimate the ${\mathcal {X}}_{p-\ell -\beta }$ norm by the stronger ${\mathcal {X}}_\mu$ norm. For real values of $\mu$, we use interpolation.

We still need to prove the result when $p-\ell -\beta =-1,$ which can only happen when $\ell =p$ and $\beta =1.$ We note that $r_{p,1}(0)=0$ and we can therefore argue as in the previous case for $\mu =0$. $\square$

#### Lemma 5.5

If the RK method satisfies Assumption 2.III and $k_0$ is the value given in Lemma 5.4, then

5.12 $$\begin{aligned} \Vert s_n(k \,A) {\mathbf {g}}(k\, A)^\top \Vert _{{\mathcal {X}}^m\rightarrow \mathcal {X}}\le C\, \rho _k(T), \end{aligned}$$

for all $k\le k_0$ and *n* such that $nk\le T$.

#### Proof

Since ${\mathbf {g}}(0)=\mathbf{0 }$, we can adapt the proof of Lemma 5.4 to each of the components of the vector-valued function ${\mathbf {g}}$. The key step is to show that $\mathbf{h}(z)^\top :=(r(z)-1)^{-1} {\mathbf {g}}(z)$ is bounded at infinity and has all its poles in the set defined in (5.10) and therefore

$$\begin{aligned} \Vert {\mathbf {h}}(k\,A)^\top \Vert _{{\mathcal {X}}^m \rightarrow {\mathcal {X}}}\le C \qquad \forall k\le k_0. \end{aligned}$$

Since the operator $s_n(k \,A) {\mathbf {g}}(k\, A)^\top$ on the left-hand side of (5.12) can be rewritten as $(r(kA)^{n+1}-I){\mathbf {h}}(k\,A)^\top$, the bound (5.12) follows readily. $\square$

When dealing with Runge–Kutta methods that do not satisfy the additional Assumption 2.III, we still have the following result:

#### Lemma 5.6

For $k_0>0$ taken as in Lemma 5.4, we can bound for all $k\le k_0$

5.13 $$\begin{aligned} \Vert r_{\ell ,\beta }(k\,A)\Vert _{{\mathcal {X}}_\mu \rightarrow {\mathcal {X}}}\le C k^{\min \{\mu ,p+1-\ell -\beta \}} \end{aligned}$$

for $\ell \le p$, $\beta \in \{0,1\}$, and $\mu \ge 0$. The constant *C* depends on *M*, $\omega$, $k_0$, $\mu$, and the RK method. The estimate (5.13) also holds for $\beta =-1$ if the method is stiffly accurate. Additionally

5.14 $$\begin{aligned} \Vert {\mathbf {g}}(k A)^\top \Vert _{{\mathcal {X}}^m\rightarrow {\mathcal {X}}}\le C \qquad \forall k\le k_0. \end{aligned}$$

#### Proof

The argument to prove (5.13) is very similar to that of Lemma 5.4. By interpolation it is clear that we just need to prove the result for any integer $\mu$ satisfying $0\le \mu \le p+1-\ell -\beta$. Consider then the rational function $q_{\ell ,\beta ,\mu }(z):=z^{-\mu }r_{\ell ,\beta }(z)$, which is bounded at infinity and has all its poles in $\sigma ({\mathcal {Q}}^{-1})$ [see (5.10)]. We can then use the same argument to prove (5.11) for this redefined new function $q_{\ell ,\beta ,\mu }$. (Note that we do not use Assumption 2.III in this argument.) Using that $r_{\ell ,\beta }(k\,A)=k^\mu q_{\ell ,\beta ,\mu }(k A) A^\mu$ in ${\text {dom}}\left( A^\mu \right)$, the result follows. Stiff accuracy of the method is used in the case $\beta =-1$ when we apply Lemma 5.3, dealing with the zeros of $r_{\ell ,-1}$.

The proof of (5.14) is a similar adaptation of the proof of Lemma 5.5. $\square$

### Estimates on discrete convolutions

The RK error will naturally induce several types of discrete convolutions that we will need to estimate separately. In all of them we will have the structure

5.15 $$\begin{aligned} \omega _0 =0, \qquad \omega _{n+1}:=r(kA)\omega _n+k\eta _n,\quad n\ge 0. \end{aligned}$$

We first deal with the simplest cases.

#### Lemma 5.7

For $nk\le T$, the sequence defined by (5.15) can be bounded by

$$\begin{aligned} \Vert \omega _n\Vert _{{\mathcal {X}}}\le n k \rho _k(T) \max _{j \le n} \Vert \eta _j\Vert _{{\mathcal {X}}} \le T \rho _k(T) \max _{j\le n} \Vert \eta _j\Vert _{{\mathcal {X}}}. \end{aligned}$$

If $\eta _n:={\mathbf {g}}(kA)^\top {\varvec{\xi }}_n$ for ${\varvec{\xi }}_n\in {\mathcal {X}}^m$, then

$$\begin{aligned} \Vert \omega _n\Vert _{{\mathcal {X}}} \le C T \rho _k(T) \max _{j\le n} \Vert {\varvec{\xi }}_n\Vert _{{\mathcal {X}}^m}. \end{aligned}$$

#### Proof

Follows by writing the recurrence (5.15) as a discrete convolution. $\square$

The next estimate is related to the consistency error of the RK method in the sense of how the RK method approximates derivatives at the stage level. We introduce the operator

5.16 $$\begin{aligned} {{\varvec{D}}}^k(y;t):=y(t+k{\mathbf {c}})-y(t){\mathbf {1}}-k {\mathcal {Q}}\dot{y}(t+k{\mathbf {c}}). \end{aligned}$$

The following well-known result about ${{\varvec{D}}}^k(y;t)$ underlies the proofs of [1, Theorem 1] and [25, Theorem 2].

#### Lemma 5.8

If $$y\in {\mathcal {C}}^{p+1}([0,T];{\mathcal {X}})$$, then

5.17 $$\begin{aligned} {{\varvec{D}}}^k(y;t)= \sum _{j=q+1}^p \frac{k^j}{j!} ({\mathbf {c}}^j-j{\mathcal {Q}}{\mathbf {c}}^{j-1}) y^{(j)}(t)+{{\varvec{R}}}^k(t), \end{aligned}$$

where

5.18 $$\begin{aligned} \Vert {{\varvec{R}}}^k(t)\Vert _{{\mathcal {X}}^m}\le C k^{p+1} \max _{t\le \tau \le t+k} \Vert y^{(p+1)}(\tau )\Vert _{{\mathcal {X}}}. \end{aligned}$$

#### Proof

Follows easily from the Taylor expansion and the order conditions (5.1a, 5.1b). $\square$

We are almost ready for the two main lemmas of this section, the first one without Assumption 2.III and the second one with it. These results and their proofs follow [1, Theorem 1 and 2], where only the case $\beta =0$ is covered.

#### Lemma 5.9

Let $y\in {\mathcal {C}}^{p+1}([0,T];{\mathcal {X}})\cap \mathcal C^p([0,T];{\mathcal {X}}_\mu )$ for some $\mu \ge 0$. Let the sequence $\omega _n$ be defined by (5.15) with $\eta _n:=k^{-1}{\mathbf {g}}(kA)^\top (k{\mathcal {Q}})^\beta {\varvec{D}}(y;t_n)$. Then there exists a constant $k_0>0$ depending only on $\omega$ from (2.2) and the RK method such that for $k \le k_0$, $\beta \in \{0,1\}$ and for $n k \le T$

$$\begin{aligned} \Vert \omega _n\Vert _{{\mathcal {X}}} \le C T \rho _k(T) k^{\min \{q+\mu +\beta ,p+\beta ,p\}} \left( \sum _{j=q+1}^p \Vert y^{(j)}\Vert _{T,\mu } +\Vert y^{(p+1)}\Vert _{T,0}\right) . \end{aligned}$$

The estimate also holds for $\beta =-1$ if the method is stiffly accurate. If $\omega =0$, then $k_0$ can be chosen arbitrarily.

#### Proof

Introduce

5.19 $$\begin{aligned} e^k_\beta (t) :={\mathbf {g}}(kA)^\top (k{\mathcal {Q}})^\beta {\varvec{D}}^k(y;t), \qquad \beta \in \{-1,0,1\}, \end{aligned}$$

and note $\eta _n=k^{-1}e^k_\beta (t_n)$. Using Lemmas 5.6 and 5.8 [recall the definition of $r_{j,\beta }$ in (5.5)], we can bound

$$\begin{aligned} \Vert e^k_\beta (t)\Vert _{{\mathcal {X}}}&\le \sum _{j=q+1}^p \frac{k^{j+\beta }}{j!} \left\| r_{j,\beta }(kA)y^{(j)}(t)\right\| _{{\mathcal {X}}} +k^\beta \Vert {\mathbf {g}}(kA)^\top {\mathcal {Q}}^\beta {{\varvec{R}}}^k(t)\Vert _{{\mathcal {X}}} \\&\lesssim \sum _{j=q+1}^p k^{\min \{\mu +j+\beta ,p+1\}} \Vert y^{(j)}(t)\Vert _{{\mathcal {X}}_\mu } + k^{\beta } \Vert {\varvec{R}}^k(t)\Vert _{{\mathcal {X}}^m}. \end{aligned}$$

By (5.18), we then have

5.20 $$\begin{aligned} \Vert e^k_\beta (t)\Vert _{{\mathcal {X}}}&\lesssim k^{1+\min \{q+\mu +\beta ,p+\beta ,p\}} \Big (\!\sum _{j=q+1}^p \Vert y^{(j)}(t)\Vert _{{\mathcal {X}}_\mu } +\max _{t\le \tau \le t+k}\Vert y^{(p+1)}(\tau )\Vert _{{\mathcal {X}}}\Big ), \end{aligned}$$

and the result then follows from Lemma 5.7. $\square$

#### Lemma 5.10

Let $y\in {\mathcal {C}}^{p+1}([0,T];{\mathcal {X}}_\mu )$ for some $\mu \ge 0$. Let the RK method satisfy Assumption 2.III. Then there exists a constant $k_0>0$ depending only on $\omega$ from (2.2) and the RK method such that the sequence $\omega _n$ defined in Lemma 5.9 satisfies for $\beta \in \{0,1\}$ and $k\le k_0$

$$\begin{aligned} \Vert \omega _n\Vert _{{\mathcal {X}}} \le C (1+T) \rho _k(T) k^{\min \{q+\mu +\beta +1,p\}} \left( \sum _{j=q+1}^{p+1} \Vert y^{(j)}\Vert _{T,\mu } \right) . \end{aligned}$$

If the method is stiffly accurate and $y\in \mathcal {C}^{p+2}([0,T];{\mathcal {X}})\cap {\mathcal {C}}^{p+1}([0,T];\mathcal {X}_\mu )$, then for $\beta =-1$

$$\begin{aligned} \Vert \omega _n\Vert _{{\mathcal {X}}} \le C (1+T) \rho _k(T) k^{\min \{q+\mu ,p\}} \left( \sum _{j=q+1}^{p+1} \Vert y^{(j)}\Vert _{T,\mu } +\Vert y^{(p+2)}\Vert _{T,0}\right) . \end{aligned}$$

If $\omega =0$, then $k_0$ can be chosen arbitrarily.

#### Proof

We will use the function $e^k_\beta$ defined in (5.19) and Abel’s summation by parts:

5.21 $$\begin{aligned} \omega _n&= \sum _{j=0}^n r(kA)^{n-j} e^k_\beta (t_j) \nonumber \\&= s_n(kA) e^k_\beta (t_0) +\sum _{j=1}^n s_{n-j}(kA)(e^k_\beta (t_j)-e^k_\beta (t_{j-1})) + e^k_\beta (t_n), \end{aligned}$$

an expression involving the rational functions $s_n$ defined in (5.6) (recall that $s_0=1$). We first apply Lemmas 5.4, 5.5 and 5.8 to estimate

$$\begin{aligned} \Vert s_n(kA) e^k_\beta (t)\Vert _{{\mathcal {X}}} & \le\sum _{j=q+1}^p \frac{k^{j+\beta }}{j!} \Vert s_n(kA)r_{j,\beta }(kA)\Vert _{\mathcal {X}_\mu \rightarrow {\mathcal {X}}} \Vert y^{(j)}(t)\Vert _{{\mathcal {X}}_\mu }\\& \quad \quad \quad \quad \quad + C k^\beta \Vert s_n(kA){\mathbf {g}}(kA)^\top \Vert _{\mathcal {X}^m\rightarrow {\mathcal {X}}} \Vert {{\varvec{R}}}^k(t)\Vert _{{\mathcal {X}}^m}\\ & \lesssim\rho _k(T) \sum _{j=q+1}^p k^{\min \{j+\mu +\beta ,p\}}\Vert y^{(j)}(t)\Vert _{{\mathcal {X}}_\mu } \\& \quad \quad \quad \quad \quad + \rho _k(T) k^{\beta +p+1} \max _{t\le \tau \le t+k} \Vert y^{(p+1)}(\tau )\Vert _{{\mathcal {X}}}\\ & \lesssim \rho _k(T) k^{1+\min \{q+\mu +\beta ,p+\beta ,p-1\}} \biggl (\sum _{j=q+1}^p \Vert y^{(j)}(t)\Vert _{{\mathcal {X}}_\mu } \\& \quad \quad \quad \quad \quad \quad \quad \quad \quad \quad \quad \quad +\max _{t\le \tau \le t+k} \Vert y^{(p+1)}(\tau )\Vert _{\mathcal {X}}\biggr ). \end{aligned}$$

Since

$$\begin{aligned} e^k_\beta (t)-e^k_\beta (t-k) ={\mathbf {g}}(kA)^\top (k{\mathcal {Q}})^\beta {{\varvec{D}}}^k(y-y(\cdot -k);t), \end{aligned}$$

and using that $\left\| y^{(j)}(t)-y^{(j)}(t-k)\right\| _{{\mathcal {X}}_{\mu }} \le k\max _{t-k\le \tau \le t+k}\left\| y^{(j+1)}\right\| _{{\mathcal {X}}_{\mu }}$, a computation analogous to the above bound, but using $y-y(\cdot -k)$ as data implies

$$\begin{aligned} &\Vert s_n(kA)(e^k_\beta (t)-e^k_\beta (t-k)\Vert _{{\mathcal {X}}} \lesssim \rho _k(T) k^{1+\min \{q+1+\mu +\beta ,p\}} \\& \quad \quad \quad \quad \quad \Biggl [ \sum _{j=q+2}^{p+1} \max _{t-k\le \tau \le t} \Vert y^{(j)}(\tau )\Vert _{{\mathcal {X}}_\mu } +\max _{t-k\le \tau \le t+k}\Vert y^{(p+2)}(\tau )\Vert _{{\mathcal {X}}}\!\Biggr ], \end{aligned}$$

and therefore

$$\begin{aligned} \sum _{j=1}^n \Vert s_{n-j}(kA)(e^k_\beta (t_j)-e^k_\beta (t_{j-1}))\Vert _{{\mathcal {X}}} \lesssim \rho _k(T) t_n k^{\min \{q+1+\mu +\beta ,p\}} \left( \sum _{j=q+2}^{p+1} \Vert y^{(j)}\Vert _{t_n,\mu } +\Vert y^{(p+2)}\Vert _{t_{n+1},0}\right) . \end{aligned}$$

Note that if $\beta \in \{0,1\}$ we can make a simpler estimate for the term originating from ${\mathbf {R}}^k$, (i.e., the one containing the highest derivative) using less regularity for *y* by not taking advantage of the difference between $y^{(p+1)}(t_j)$ and $y^{(p+1)}(t_{j-1})$ and thus end up requiring less regularity. Using the estimate (5.20) for the last term in (5.21), we have thereby already derived estimates for all three terms in (5.21). $\square$

## Proofs

The two different cases (with or without Assumption 2.III) will be collected by using the parameter

6.1 $$\begin{aligned} \alpha := {\left\{ \begin{array}{ll} 1, &{} \text{ if } \text{ Assumption }~2.III \,\text{ holds }, \\ 0, &{} \text{ otherwise }. \end{array}\right. } \end{aligned}$$

### Proof of Theorem 3.4

Recall that *u* solves (2.1) with $u(0)=0$. The functions $\varXi$ and *F* are the given boundary and volume data. If $\varGamma :=\partial ^{-1} \varXi$ and $H:=\partial ^{-1}F$, then $x=\partial ^{-1} u$ solves

6.2 $$\begin{aligned} \dot{x}(t)=A_\star x(t)+H(t), \quad t>0, \qquad Bx(t)=\varGamma (t), \qquad x(0)=0. \end{aligned}$$

On the other hand, $\{ X^k_n\}=(\partial ^k)^{-1} \{ U^k_n\}$ solves by Proposition 4.6:

6.3a $$\begin{aligned} X_n^k =&{\mathbf {1}}x_n^k + k({\mathcal {Q}}\otimes A_\star ) X^k_n+ k{\mathcal {Q}}H^k_n, \end{aligned}$$

6.3b $$\begin{aligned} ({{\mathcal {I}}}\otimes B) X_n^k =&\varGamma _n^k, \end{aligned}$$

6.3c $$\begin{aligned} x_{n+1}^k =&x_n^k + k({\mathbf {b}}^\top \otimes A_\star ) X^k_n + k {\mathbf {b}}^\top H^k_n. \end{aligned}$$

Before we can estimate the difference between the functions *x* and $x_n^k$, we need one final lemma.

#### Lemma 6.1

Let *x* solve

6.4 $$\begin{aligned} \dot{x}(t)=A_\star x(t)+H(t), \quad t>0, \qquad Bx(t)=\varGamma (t), \qquad x(0)=0. \end{aligned}$$

Assume that for some $\mu \ge 0$ we have

$$\begin{aligned} x\in {\mathcal {C}}^{p+1}([0,T];{\mathcal {X}}_\mu ), \quad H \in \mathcal {C}^p([0,T];{\mathcal {X}}_\mu ), \quad {\mathscr {E}}\varGamma \in \mathcal {C}^p([0,T];{\mathcal {X}}_\mu ). \end{aligned}$$

Then $$x - {\mathscr {E}}\varGamma \in {\mathcal {C}}^p([0,T];\mathcal X_{\mu +1})$$.

#### Proof

We set $y:=x - {\mathscr {E}}\varGamma$. By assumption we have $y \in {{\mathcal {C}}}^p([0,T];{\mathcal {X}}_{\mu })$ and $B\left( x- {\mathscr {E}}\varGamma \right) =0$. Since $x \in {\text {dom}}(A_\star )$ and ${\text {range }}{{\mathscr {E}}}\subset {\text {dom}}(A_\star )$ this implies $y(t) \in {\text {dom}}(A)$ for all $t \in [0,T]$. We further calculate using (6.4) and ${\text {range }}{\mathscr {E}}\subseteq {\text {ker}}(I-A_\star)$:

$$\begin{aligned} Ay&= A_{\star } x - A_{\star } {\mathscr {E}}\varGamma = {\dot{x}} - H - {\mathscr {E}}\varGamma . \end{aligned}$$

Each of the terms is assumed in ${\mathcal {C}}^p([0,T];\mathcal X_{\mu })$, thus $y \in {\mathcal {C}}^p([0,T];{\mathcal {X}}_{\mu +1})$. $\square$

We will need the sequences $\{ \gamma _n^k\}$ and $\{ h_n^k\}$ with the scalar parts of the computations of $\{\varGamma ^k_n\}$ and $\{H^k_n\}$ respectively, namely (see Lemma 2.6),

6.5a $$\begin{aligned} \gamma ^k_0:=0,&\qquad \gamma ^k_n=\gamma ^k_{n-1}+k{\mathbf {b}}^\top \varXi (t_n+k{\mathbf {c}}), \end{aligned}$$

6.5b $$\begin{aligned} h^k_0:=0,&\qquad h^k_n=h^k_{n-1}+k{\mathbf {b}}^\top F(t_n+k{\mathbf {c}}). \end{aligned}$$

We then consider

$$\begin{aligned} \varDelta _n^k:=({{\mathcal {I}}}\otimes {\mathscr {E}})(\varGamma (t_n+k{\mathbf {c}})-\varGamma _n^k), \qquad \delta _n^k:={\mathscr {E}}(\varGamma (t_n)-\gamma _n^k). \end{aligned}$$

Using (6.5a), the definition $\varGamma ^k=(\partial ^k)^{-1} \varXi$, and (2.13), we can write

6.6 $$\begin{aligned} \varDelta _n^k-{\mathbf {1}}\delta ^k_n&=({{\mathcal {I}}} \otimes {\mathscr {E}}) \varGamma (t_n+k{\mathbf {c}})-{\mathbf {1}}{\mathscr {E}}\varGamma (t_n)-k{\mathcal {Q}}\otimes {\mathscr {E}}{\dot{\varGamma }}(t_n+k{\mathbf {c}}) \nonumber \\&={{\varvec{D}}}^k({\mathscr {E}}\varGamma ;t_n). \end{aligned}$$

Lemma 5.2 (take $f={\mathscr {E}}\varXi$ for the first three inequalities and $f=F$ for the last one) proves that

6.7a $$\begin{aligned} \Vert \delta ^k_n\Vert _{{\mathcal {X}}} \le&C T k^p \Vert {\mathscr {E}}\varXi ^{(p)}\Vert _{T,0}, \end{aligned}$$

6.7b $$\begin{aligned} \Vert \delta ^k_n-\delta ^k_{n-1}\Vert _{{\mathcal {X}}} \le&C k^{p+1} \Vert {\mathscr {E}}\varXi ^{(p)}\Vert _{T,0}, \end{aligned}$$

6.7c $$\begin{aligned} \Vert k{\mathbf {b}}^\top \varDelta ^k_n\Vert _{{\mathcal {X}}} \le&C k^{p+1} ( \Vert {\mathscr {E}}\varXi ^{(p-1)}\Vert _{T,0}+T \Vert {\mathscr {E}}\varXi ^{(p)}\Vert _{T,0}), \end{aligned}$$

6.7d $$\begin{aligned} \Vert H(t_n)-h_n^k\Vert _{{\mathcal {X}}} \le&C T k^p \Vert F^{(p)}\Vert _{T,0}. \end{aligned}$$

The error analysis is derived by tracking the evolution of the following differences

$$\begin{aligned} E_n^k := x(t_n + k {\mathbf {c}})-X^k_n-\varDelta ^k_n \in ({\text {dom}} (A))^m, \qquad e^k_n := x(t_n)-x^k_n -\delta ^k_n, \end{aligned}$$

(compare (6.2) and (6.3b) to see the vanishing boundary condition for $E_n^k$) and note that by (6.7a)

$$\begin{aligned} \Vert x(t_n)-x^k_n\Vert _{{\mathcal {X}}} \le \Vert e^k_n\Vert _{{\mathcal {X}}}+ C T k^p \Vert {\mathscr {E}}\varXi ^{(p)}\Vert _{T,0}, \end{aligned}$$

which shows that we only need to estimate $e^k_n$ to prove Theorem 3.4.

We start with the observation that *x* solves the following equation, as can be easily derived from Eq. (6.2):

6.8 $$\begin{aligned} x(t_n + k {\mathbf {c}})&= {\mathbf {1}}x(t_n) + k {\mathcal {Q}}\otimes A_{\star }x(t_n+k {\mathbf {c}}) \nonumber \\&\quad + x(t_n+k {\mathbf {c}}) - k {\mathcal {Q}}{\dot{x}}(t_n+k {\mathbf {c}}) + k {\mathcal {Q}}H(t_n+k{\mathbf {c}}) - {\mathbf {1}}x(t_n)\nonumber \\&= {\mathbf {1}}x(t_n) + k {\mathcal {Q}}\otimes A_{\star }x(t_n+k {\mathbf {c}}) + k{\mathcal {Q}}H(t_n+k{\mathbf {c}}) + {{\varvec{D}}}^{k}(x,t_n) . \end{aligned}$$

Recalling that Assumption 2.I included the hypothesis $\mathrm {range}\,{\mathscr {E}}\subset \ker (I-A_\star )$, we have $({\mathcal {Q}}\otimes A_\star )\varDelta ^k_n={\mathcal {Q}}\varDelta ^k_n$. Combining (6.8) and (6.3a), we get

$$\begin{aligned} E^k_n={\mathbf {1}}e^k_n+ k({\mathcal {Q}}\otimes A)E^k_n + {{\varvec{D}}}^k(x,t_n) - k {\mathcal {Q}}\big (H^k_n - H(t_n + k {\mathbf {c}})\big ) +{\mathbf {1}}\delta ^k_n-\varDelta ^k_n+k{\mathcal {Q}}\varDelta ^k_n. \end{aligned}$$

Naive estimation of the terms ${{\varvec{D}}}^k(x,t_n)$ and $\varDelta ^k_n - {\mathbf {1}}\delta ^k_n$ would yield convergence rates similar to Propositions 3.1 and 3.2. In order to get an increased rate, as stated in Theorem 3.4, we combine these two terms using the function $Y(t):=x(t) - {\mathscr {E}}\varGamma (t)$. Lemma 6.1 and the assumptions of Theorem 3.4 ensure $Y \in {\mathcal {C}}^{p+\alpha }([0,T];{\mathcal {X}}_{\mu +1}) \cap {\mathcal {C}}^{p+1}([0,T];{\mathcal {X}})$.

We can thus further simplify

6.9 $$\begin{aligned} E^k_n&= {\mathbf {1}}e^k_n+ k({\mathcal {Q}}\otimes A)E^k_n + {{\varvec{D}}}^k(x,t_n) - {{\varvec{D}}}^k({\mathscr {E}}\varGamma ,t_n) \nonumber \\&\quad + k {\mathcal {Q}}{{\varvec{D}}}^k(H,t_n) - k {\mathcal {Q}}{\mathbf {1}}\big ( h^k_h - H(t_n)\big ) +k{\mathcal {Q}}\varDelta ^k_n \nonumber \\&= {\mathbf {1}}e^k_n+ k({\mathcal {Q}}\otimes A)E^k_n + {{\varvec{D}}}^k(Y,t_n) \nonumber \\ &\quad +k {\mathcal {Q}}{{\varvec{D}}}^k(H,t_n) - k {\mathcal {Q}}{\mathbf {1}}\big (h^k_h - H(t_n)\big ) +k{\mathcal {Q}}\varDelta ^k_n. \end{aligned}$$

This then immediately gives (recall (5.7) for the definition of ${\mathbf {g}}$)

6.10 $$\begin{aligned}&k({\mathbf {b}}^\top \otimes A)E^k_n = \nonumber \\&\qquad {\mathbf {g}}(kA)^\top \left[ {\mathbf {1}}e^k_n + {{\varvec{D}}}^k(Y,t) + k {\mathcal {Q}}{{\varvec{D}}}^k(H,t_n) - k {\mathcal {Q}}{\mathbf {1}}\big (h^k_h - H(t_n)\big ) +k{\mathcal {Q}}\varDelta ^k_n\right] . \end{aligned}$$

It is easy to see from (6.2) that *x* satisfies

$$\begin{aligned} x(t_{n+1}) &=x(t_n) + k {\mathbf {b}}^\top \otimes A_{\star } x(t_n + k \,{\mathbf {c}}) \\&\quad + \left[ x(t_{n+1}) - x(t_n) - k {\mathbf {b}}^\top {\dot{x}}(t_{n}+ k{\mathbf {c}}) + k {\mathbf {b}}^\top H(t_n+k {\mathbf {c}})\right] . \end{aligned}$$

Subtracting (6.3c) from this, inserting (6.10), using that $({\mathbf {b}}^\top \otimes A_\star )\varDelta ^k_n={\mathbf {b}}^\top \varDelta ^k_n$, and setting

$$\begin{aligned} \varphi ^k_n:=\left[ x(t_{n+1}) -x(t_n) - k {\mathbf {b}}^\top {\dot{x}}(t_{n}+k{\mathbf {c}})\right] +k {\mathbf {b}}^\top (H(t_n + k{\mathbf {c}}) - H^k_n), \end{aligned}$$

we have

$$\begin{aligned} e^k_{n+1}&= e^k_n+k({\mathbf {b}}^\top \otimes A)E^k_n + k({\mathbf {b}}^\top \otimes A_\star ) \varDelta ^k_n+\delta ^k_n-\delta ^k_{n+1} + \varphi ^k_n\\&= r(kA)e^k_n +{\mathbf {g}}(kA)^\top (k{\mathcal {Q}})\varDelta ^k_n +{\mathbf {g}}(kA)^\top {{\varvec{D}}}^k(Y,t_n) \\&\quad + {\mathbf {g}}(kA)^\top (k{\mathcal {Q}}){{\varvec{D}}}^k(H,t_n) - {\mathbf {g}}(kA)^\top (k{\mathcal {Q}}) {\mathbf {1}}\big (h^k_h - H(t_n)\big ) \\&\quad + k{\mathbf {b}}^\top \varDelta ^k_n+\delta ^k_n-\delta ^k_{n+1} +\varphi ^k_n. \end{aligned}$$

What is left is the careful combination of terms so that we can bound everything using Lemmas 5.7, 5.9, and 5.10 by writing

$$\begin{aligned} e^k_{n+1}-r(kA) e_n^k =\,&{\mathbf {g}}(kA)^\top ({{\varvec{D}}}^k(Y;t_n)) +{\mathbf {g}}(kA)^\top (k{\mathcal {Q}}) ({{\varvec{D}}}^k({\mathscr {E}}\varGamma + H;t_n)) \\&+{\mathbf {g}}(kA)^\top {\mathcal {Q}}{\mathbf {1}}\, k \big (\delta _n^k - \big (h^k_h - H(t_n)\big )\big ) \\&+ k{\mathbf {b}}^\top \varDelta _n^k + (\delta _n^k-\delta _{n+1}^k) + \varphi ^k_n. \end{aligned}$$

Since the above recurrence defining $\{e^k_n\}$ is linear as a function of the right-hand side, we can estimate its norm by adding the effects of each of the terms. In the order in which they appear in the last expression, we use: Lemmas 5.9–5.10 with $\beta =0$, but noting that $Y(t) \in {\text {dom}}(A^{\mu +1})$; Lemmas 5.9–5.10 with $\beta =1$; Lemma 5.7 combined with (6.7a) and (6.7d); Lemma 5.7 combined with (6.7c) and (6.7b); for the first term of $\varphi ^k_h$ we use Lemma 5.7 combined with Lemma 5.2 with $f:={\dot{x}}$. Finally, for the second contribution to $\varphi ^k_h$, we use (5.4c).

Combined, these results give

$$\begin{aligned} \Vert e_n^k\Vert _{{\mathcal {X}}} \le&C (T+ \alpha) \rho _k(T) k^{\min \{q+\mu +1+\alpha ,p\}} \Big (\sum _{j =q+1}^{p} \Vert Y^{(j)}\Vert _{T,\mu +1} +\Vert Y^{(p+1)}\Vert _{T,\alpha (\mu +1)}\Big ) \\&+ C (T+ \alpha) \rho _k(T) k^{\min \{q+\mu +1+\alpha ,p\}} \Big (\sum _{j =q+1}^{p} \Vert ({\mathscr {E}}\varGamma )^{(j)}\Vert _{T,\mu } +\Vert ({\mathscr {E}}\varGamma )^{(p+1)}\Vert _{T,\alpha \mu }\Big ) \\&+ C (T+ \alpha) \rho _k(T) k^{\min \{q+\mu +1+\alpha ,p\}} \Big (\sum _{j =q+1}^{p} \Vert H^{(j)}\Vert _{T,\mu } +\Vert H^{(p+1)}\Vert _{T,\alpha \mu }\Big ) \\&+ CT^2 \rho _k(T) k^{p+1} \Vert {\mathscr {E}}\varXi ^{(p)}\Vert _{T,0} + C T^2 \rho _k(T) k^{p+1} \Vert {F^{(p)}}\Vert _{T,0}\\&+ CT\rho _k(T) k^{p+1} \big ( \Vert {\mathscr {E}}\varXi ^{(p-1)}\Vert _{T,0}+T \Vert {\mathscr {E}}\varXi ^{(p)}\Vert _{T,0}\big ) \\&+ C T\rho _k(T) k^{p+1} \big ( \Vert H^{(p-1)}\Vert _{T,0}+T \Vert H^{(p)}\Vert _{T,0}\big ). \end{aligned}$$

If we apply Lemma 6.1 to bound the ${\mathcal {X}}_{\mu +1}$-norm, we arrive at the stated estimate.

### Proof of Theorem 3.5

This proof is very similar to the one of Theorem 3.4 but slightly simpler. We will point out the main steps of the proof. We first focus on showing the estimate for $v-v^k$. Note that we use the simple form of $\partial ^k$ for stiffly accurate RK methods given in Lemma 2.7. We define $G:=\dot{F}$ and $\varTheta :={\dot{\varXi }}$ so that $v=\dot{u}$ satisfies

$$\begin{aligned} \dot{v}(t)=A_\star v(t)+G(t), \quad t>0, \qquad Bv(t)=\varTheta (t), \qquad v(0)=0. \end{aligned}$$

Its RK approximation

6.11a $$\begin{aligned} {\widetilde{V}}_n^k =& \, {\mathbf {1}}{\widetilde{v}}_n^k + k({\mathcal {Q}}\otimes A_\star ){\widetilde{V}}^k_n + k{\mathcal {Q}}G(t_n+k{\mathbf {c}}), \end{aligned}$$

6.11b $$\begin{aligned} ({{\mathcal {I}}}\otimes B) {\widetilde{V}}_n^k =& \, \varTheta (t_n+k{\mathbf {c}}), \end{aligned}$$

6.11c $$\begin{aligned} {\widetilde{v}}_{n+1}^k =& \, {\widetilde{v}}_n^k + k({\mathbf {b}}^\top \otimes A_\star ) {\widetilde{V}}^k_n + k {\mathbf {b}}^\top G(t_n + k {\mathbf {c}}), \end{aligned}$$

and $\{V_n^k\}=\partial ^k \{U_n^k\}$ satisfies (see Proposition 4.5 and Lemma 2.7, where we use stiff accuracy of the RK scheme, and recall that $\{G^k_n\}=\partial ^k \{F(t_n+k{\mathbf {c}})\}$ and $\{\varTheta ^k_n\}=\partial ^k\{ \varXi (t_n+k{\mathbf {c}})\}$)

6.12a $$\begin{aligned} V_n^k =\,&{\mathbf {1}}v_n^k + k({\mathcal {Q}}\otimes A_\star ) V^k_n+ k{\mathcal {Q}}G^k_n, \end{aligned}$$

6.12b $$\begin{aligned} ({{\mathcal {I}}}\otimes B) V_n^k =\,&\varTheta _n^k=k^{-1}{\mathcal {Q}}^{-1}(\varXi (t_n+k{\mathbf {c}})-{\mathbf {1}}\varXi (t_n)), \end{aligned}$$

6.12c $$\begin{aligned} v_{n+1}^k =\,&v_n^k + k({\mathbf {b}}^\top \otimes A_\star ) V^k_n + k {\mathbf {b}}^\top G_n^k. \end{aligned}$$

Let then

$$\begin{aligned} \varDelta ^k_n:=({\mathcal {I}}\otimes {\mathscr {E}})(\varTheta _n^k-\varTheta (t_n+k{\mathbf {c}})) =k^{-1}{\mathcal {Q}}^{-1} {\varvec{D}}^k({\mathscr {E}}\varXi ;t_n) \end{aligned}$$

and [note (6.11b) and (6.11c)]

$$\begin{aligned} E^k_n := V^k_n-{\widetilde{V}}^k_n-\varDelta ^k_n \in ({\text {dom}} (A))^m, \qquad e^k_n := v^k_n-{\widetilde{v}}^k_n. \end{aligned}$$

By (6.11a) and (6.12a), using that $({\mathcal {Q}}\otimes A_\star )\varDelta _n^k={\mathcal {Q}}\varDelta ^k_n$ (assumption on the lifting) and Lemma 2.7 to represent $G_n^k$, we have

$$\begin{aligned} k({\mathbf {b}}^\top \otimes A) E^k_n ={\mathbf {g}}(kA)^\top ({\mathbf {1}}e_n^k-\varDelta _n^k+k{\mathcal {Q}}\varDelta _n^k+{{\varvec{D}}}^k(F;t_n)) \end{aligned}$$

and therefore, from (6.11c) and (6.12c)

6.13 $$\begin{aligned} e^k_{n+1}& =\, {} r(kA) e^k_n -{\mathbf {g}}(kA)^\top (k{\mathcal {Q}})^{-1}{\varvec{D}}^k({\mathscr {E}}\varXi ;t_n) +{\mathbf {g}}(kA)^\top {{\varvec{D}}}^k({\mathscr {E}}\varXi +F;t_n) \nonumber \\&\quad + k {\mathbf {b}}^\top {\mathcal {Q}}^{-1} D^k({\mathscr {E}}\varXi +F;t_n). \end{aligned}$$

The final term can be shown to be of order ${\mathcal {O}}(k^{p+1})$ by combining (5.17) with (5.2b) and (5.3). We then use Lemmas 5.9 and 5.10 with $\beta =-1$ and $\beta =0$ as well as Lemma 5.7 to bound

$$\begin{aligned} \|e_{n}^{k}\|{}_{{\mathcal{X}}} &\le {\text{C}}(T+ \alpha) \rho _{k} (T)k^{{\alpha - 1 + \min \{ q + \mu ,p\} }} \left( {\sum\limits_{{j = q + 1}}^{{p + \alpha }} \|{{\mathscr{E}}{\varXi}^{{(j)}}\| {}_{{T,\mu }} } + \|{\mathscr{E}}{\varXi}^{{(p + 1 + \alpha )}}\| {}_{{T,0}} } \right) \\ &\quad + C(T+ \alpha) \rho _{k} (T)k^{{\alpha - 1 + \min \{ q + \mu ,p\} }} \left( {\sum\limits_{{j = q + 1}}^{{p + \alpha }} \|{{}F^{{(j)}} \|{}_{{T,\mu }} } + {}\|F^{{(p + 1 + \alpha )}} \| {}_{{T,0}} } \right). \end{aligned}$$

Finally Propositions 3.1 and 3.2 are used to bound

6.14 $$\begin{aligned} \Vert v(t_n)-{\widetilde{v}}_n^k\Vert _{{\mathcal {X}}} \le C (T+ \alpha) \rho _k(T)k ^{\min \{ q+\mu +\alpha ,p\}} \Big ( \sum _{\ell =q+2}^{p+1+\alpha } \Vert u^{(\ell )}\Vert _{T,\mu } + \Vert u^{(p+2)}\Vert _{T,0}\Big ). \end{aligned}$$

The estimate involving $A_{\star }u$ can be proved as an easy corollary of the estimate on *v*. Since the last stage of a stiffly accurate method is the step, we have that (4.8a) implies that

$$\begin{aligned} A_\star u^k_n=v^k_n-F(t_n) \end{aligned}$$

and therefore

$$\begin{aligned} A_\star u(t_n)-A_\star u_n^k=v(t_n)-v^k_n. \end{aligned}$$

## Maximal dissipative operators in Hilbert space

In this short section we summarize some results that show that the hypotheses on the abstract equation and its discretization are simpler for maximal dissipative operators on Hilbert spaces. These results are well-known and will be needed when applying the theory developed in the previous sections to some model problems in Sect. 8.

If *A* is maximal dissipative in the Hilbert space $\mathcal X$, i.e.,

$$\begin{aligned} \mathrm {Re} \langle Ax,x\rangle _{{\mathcal {X}}} \le 0 \qquad \forall x\in {\text {dom}}(A), \end{aligned}$$

and if $A-I:{\text {dom}}(A)\rightarrow {\mathcal {X}}$ is invertible with bounded inverse, then the constants in Proposition 2.1 can be chosen as $M=1$ and $\omega =0$. In this case *A* generates a contraction semigroup in ${\mathcal {H}}$. See [26, Sect. 1.4].

In particular, if the RK method satisfies Assumption 2.II and

7.1 $$\begin{aligned} \sigma (A)\subset \{ z\,:\, \mathrm {Re}\,z\le 0\}, \end{aligned}$$

then the Eq. (2.7a–2.7c) [or equivalently (2.8a–2.8c)], defining the RK approximation of (2.1a–2.1c) are uniquely solvable for any $k>0$ (apply Lemma 2.4 with $\mathcal S=k^{-1}{\mathcal {Q}}^{-1}$). The following lemma gives a bound for $\rho _k(T)$ in this specific setting.

### Lemma 7.1

(Discrete stability) Let $A$ be a linear, maximal dissipative operator on a Hilbert space ${\mathcal {H}}$. For *A*-stable Runge–Kutta methods and arbitrary $k>0$, we can bound

7.2 $$\begin{aligned} \left\| r(k \,A)\right\| _{{\mathcal {H}} \rightarrow {\mathcal {H}}}\le 1, \end{aligned}$$

and therefore $\rho _k(T)\le 1$ for all *k* and $T>0$.

### Proof

Let $c(z):=(z+1)/(z-1)$, and note that $c(A)=(A+I)(A-I)^{-1}$ is well defined and since

$$\begin{aligned} \Vert (A+I)x\Vert ^2-\Vert (A-I)x\Vert ^2=4\mathrm {Re}\,\langle Ax,x\rangle \le 0 \qquad \forall x\in {\text {dom}}(A), \end{aligned}$$

it is clear that $\Vert c(A)\Vert _{{\mathcal {H}} \rightarrow {\mathcal {H}}}\le 1.$ Consider now the rational function $q:=r\circ c$. Since *c* maps *B*(0; 1) bijectively into $\{ z:\mathrm {Re}\,z<0\}$ and *r* maps the latter set to *B*(0; 1) (this is A-stability), it follows that $q:B(0;1)\rightarrow B(0;1)$. Since $\sigma (c(A))\subset \overline{B(0;1)}$ and *c*(*A*) is bounded, we can define *q*(*c*(*A*)) and show (use a classical result of von Neumann [37, Section 4] or [30, Chapter XI, Section 154]) that $\Vert q(c(A))\Vert _{{\mathcal {H}} \rightarrow {\mathcal {H}}}\le 1$.

Finally, using that $c(c(z))=z$ for all *z*, it follows that $r=q\circ c$. It is then an easy computation to prove that $r(A)=q(c(A))$. (We remark that this equality can also be proved using functional calculus.) $\square$

In Propositions 3.1 and 3.2, if *A* is maximal dissipative, $k_0$ can be chosen arbitrarily. In Lemma 5.4, if $A$ is maximal dissipative, $k_0$ can be chosen arbitrarily.

## Applications

In this section $\varOmega$ is a bounded Lipschitz open set in ${\mathbb {R}}^d$ ($d=2$ or 3) with boundary $\varGamma$.

We use the usual (fractional) Sobolev spaces $H^s(\varOmega )$ for $s\ge 0$ and introduce the space $H^1_{\varDelta }(\varOmega ):=\{ u \in H^1(\varOmega ): \varDelta u \in L^2(\varOmega ) \}$. On the boundary $\varGamma$, we also consider Sobolev spaces $H^s(\varGamma )$ and their duals $H^{-s}(\varGamma )$. Details can, for example be found in [22].

For the trace operators, we make the convention that the index $+$ relates to exterior and − means the trace is taken from the interior of $\varOmega$. For example, the two bounded surjective trace operators $\gamma ^\pm :H^1({\mathbb {R}}^d\setminus \varGamma )\rightarrow H^{1/2}(\varGamma )$ denote the trace from ${\mathbb {R}}^d\setminus {\overline{\varOmega }}$ and $\varOmega$, respectively. and we will denote $H^{-1/2}(\varGamma )$ for the dual of the trace space. The angled bracket $\langle \,\cdot \,,\,\cdot \,\rangle _\varGamma$ will be used for the $H^{-1/2}(\varGamma )\times H^{1/2}(\varGamma )$ duality pairing and $(\cdot ,\cdot )_{{\mathbb {R}}^d}$ will be used for the inner product in $L^2({\mathbb {R}}^d)$ and $\big [L^2({\mathbb {R}}^d)\big ]^d$. We will also use the normal traces $\gamma ^\pm _\nu : H({\text {div}},{\mathbb {R}}^d \setminus \varGamma )\rightarrow H^{-1/2}(\varGamma )$ and the normal derivative operators $\partial _\nu ^\pm$. Here we make the convention that the normal derivative points out of $\varOmega$ for both interior and exterior trace.

We note that the applications in this section are chosen for their simplicity. More complicated applications, also involving full discretizations by convolution quadrature and boundary elements of systems of time domain boundary integral equations can be found in [29] and [27].

### Boundary integral equations and convolution quadrature

In this section, we give a very brief introduction to boundary integral equations and their discretization using convolution quadrature. In that way, we can later easily state our methods for both the heat and wave equations in a concise and unified language. We present the result mostly formally, but note that they can be made rigorous under mild assumptions on the appearing functions. This theory can be found in most monographs on boundary element methods, see e.g. [22, 32, 33] or [31].

For $s \in {\mathbb {C}}_{+}$, we consider solutions $u \in H^1({\mathbb {R}}^d\setminus \varGamma )$ to the Helmholtz equation

$$\begin{aligned} -\varDelta u + s^2 u =0 \qquad \text {in } {\mathbb {R}}^d \setminus \varGamma . \end{aligned}$$

For this problem, the fundamental solution is given by

$$\begin{aligned} \varPhi (z;s):={\left\{ \begin{array}{ll} \frac{i}{4} H_0^{(1)}\left( is \left| z\right| \right) , &{} \text { for }d=2, \\ \frac{e^{-s\left| z\right| }}{4 \pi \left| z\right| }, &{} \text { for }d = 3, \end{array}\right. } \end{aligned}$$

where $H_0^{(1)}$ denotes the first kind Hankel function of order 0. Using the representation formula, *u* can be rewritten using only its boundary data:

8.1 $$\begin{aligned} u(x)= S(s) \llbracket \partial _{\nu } u\rrbracket - D(s)\llbracket \gamma u \rrbracket , \end{aligned}$$

where the single layer and double layer potentials are given by

$$\begin{aligned} \left( S(s) \varphi \right) \left( x\right)&:=\int _{\varGamma }{\varPhi (x-y;s) \varphi (y) \;dy}, \\ \left( D(s) \psi \right) \left( x\right)&:=\int _{\varGamma }{\partial _{\nu(y)}\varPhi (x-y;s) \psi (y) \;dy}, \end{aligned}$$

and the expressions $\llbracket \gamma u \rrbracket :=\gamma ^- u - \gamma ^+ u$ and $\llbracket \partial _{\nu } u\rrbracket := \partial _{\nu}^- u - \partial _{\nu}^+u$ denote the jump of the trace of *v* and normal derivative across $\varGamma$.

We note that both $S(s) \lambda$ and $D(s) \psi$ solve the Helmholtz equation for any given densities $\lambda \in H^{-1/2}(\varGamma )$ and $\psi \in H^{1/2}(\varGamma )$.

We will need the following four boundary integral operators:

8.2 $$\begin{aligned} V(s):=\gamma ^\pm S(s), \quad K(s):=\frac{1}{2}(\gamma ^+ S(s) + \gamma ^- S(s)), \end{aligned}$$

8.3 $$\begin{aligned} K^t(s):=\frac{1}{2}(\partial _\nu ^+ D(s) + \partial _\nu ^- D(s)),\quad W(s):=- \partial ^{\pm }_\nu D(s). \end{aligned}$$

When solving problems in the time domain, we can leverage our knowledge of the Helmholtz equation using the Laplace transform ${\mathscr {L}}$. For an operator valued analytic function *F* with ${\text {dom}}(F) \supset {\mathbb {C}}_{+}$, we can then define the convolution operator $F(\partial ):={\mathscr {L}}^{-1} \circ \mathrm F \circ {\mathscr {L}}$, where ${\mathscr {L}}$ is the Laplace transform in the sense of causal distributions. (Precise definitions can be found in [31, Chapter 3] and [21]).

Given a Runge–Kutta method, it is then easy to define the convolution quadrature approximation to such operators, as was introduced in [19]. We just replace the Laplace transform by the *Z*-transform $\mathcal {Z}$ and *s* with the function $\delta /k$, i.e., we define:

$$\begin{aligned} F(\partial ^k) g:={\mathscr {Z}}^{-1} \left( F\bigg (\frac{\delta (\cdot)}{k}\bigg ) {\mathscr {Z}}\left[ g\right] \right) , \end{aligned}$$

where *g* denotes a sequence in the shared domain of *F*(*s*), and $k>0$ denotes the step size. The matrix-valued function $z \mapsto F(\frac{\delta (z)}{k})$ is defined using the Riesz-Dunford calculus, but can be computed in practice by diagonalizing the argument.

#### Remark 8.1

We note that our use of the notation $\partial ^k$ and $(\partial ^k)^{-1}$ is consistent with this definition by using the functions $F(s):=s$ and $F(s):=s^{-1}$.

### An exotic transmission problem

In this section we show how to apply Theorems 3.4 and 3.5 to a transmission problem in free space associated to the infinitesimal generator of a group of isometries (both $\pm A$ are maximal dissipative) with some exotic transmission conditions which impose partial observation of a trace. In Sect. 8.3 we will explain how this problem is related to a boundary integral representation of a scattering problem and how the current results yield the analysis of a fully discrete method for that integral representation. We keep the presentation brief. For more details and exemplary applications we refer to [13].

Let $Y_h$ be a closed subspace of $H^{1/2}(\varGamma )$ (in practice it will be finite-dimensional) and consider the spaces

8.4a $$\begin{aligned} {\mathbf {H}}(\mathrm {div},{\mathbb {R}}^d\setminus \varGamma ) :=&\{{\mathbf {w}} \in L^2({\mathbb {R}}^d\setminus \varGamma )^d\,:\, \nabla \cdot {\mathbf {w}}\in L^2({\mathbb {R}}^d\setminus \varGamma )\}, \end{aligned}$$

8.4b $$\begin{aligned} \mathrm {V}_h :=&\{ v\in H^1({\mathbb {R}}^d\setminus \varGamma )\,:\, \llbracket \gamma v \rrbracket \in Y_h\}, \end{aligned}$$

8.4c $$\begin{aligned} {\mathbf {W}}_h :=&\{ {\mathbf {w}}\in \mathbf {H}(\mathrm {div},{\mathbb {R}}^d\setminus \varGamma ) \,:\, \langle \gamma _{\nu}^- {\mathbf {w}}, \mu _h \rangle _{\varGamma } = 0 \; \quad \forall \mu _h \in Y_h \}, \end{aligned}$$

8.4d $$\begin{aligned} {\mathbf {W}}_h^0:=&{\mathbf {W}}_h\cap {\mathbf {H}}(\mathrm {div},{\mathbb {R}}^d) \end{aligned}$$

8.4e $$\begin{aligned} =&\{ {\mathbf {w}}\in {\mathbf {H}}(\mathrm {div},{\mathbb {R}}^d)\,:\, \langle \gamma _{\nu}^- {\mathbf {w}}, \mu _h \rangle _{\varGamma } = 0 \;\quad \forall \mu _h \in Y_h\}. \end{aligned}$$

The condition $\llbracket \gamma v \rrbracket \in Y_h$ is equivalent to

8.5 $$\begin{aligned} (\nabla \cdot {\mathbf {w}},v)_{{\mathbb {R}}^d\setminus \varGamma } +({\mathbf {w}},\nabla v)_{{\mathbb {R}}^d}=0 \qquad \forall {\mathbf {w}}\in {\mathbf {W}}_h^0. \end{aligned}$$

We then set

$$\begin{aligned} {\mathcal {X}}:=L^2({\mathbb {R}}^d \setminus \varGamma )\times L^2({\mathbb {R}}^d\setminus \varGamma )^d, \qquad {\mathcal {V}}:=\mathrm V_h\times {\mathbf {W}}_h, \qquad {\mathcal {M}}:=H^{-1/2}(\varGamma ). \end{aligned}$$

In ${\mathcal {X}}$ we use the natural inner product, in ${\mathcal {V}}$ we use the norm of $H^1({\mathbb {R}}^d\setminus \varGamma )\times \mathbf{H}(\mathrm {div},{\mathbb {R}}^d\setminus \varGamma )$, and in ${\mathcal {M}}$ we use the usual norm. We will define $A_\star :{\text {dom}}\left( A_\star \right) ={\mathcal {V}}\rightarrow {\mathcal {X}}$ and $B:{\mathcal {V}} \rightarrow {\mathcal {M}}$ by

$$\begin{aligned} A_\star (v,{\mathbf {w}}):=(\nabla \cdot {\mathbf {w}},\nabla v), \qquad B(v,{\mathbf {w}}):=\gamma _{\nu}^- {\mathbf {w}} -\gamma _{\nu}^+ {\mathbf {w}}, \end{aligned}$$

understanding that $A_\star$ can also be extended to $H^1({\mathbb {R}}^d\setminus \varGamma )\times \mathbf {H}(\mathrm {div},{\mathbb {R}}^d\setminus \varGamma )$. As we did in Assumption 2.I, we consider ${\text {dom}} (A)=\ker B =\mathrm V_h\times {\mathbf {W}}_h^0$ and define *A* as the restriction of $A_\star$ to this subset.

#### Proposition 8.2

The operators $\pm A$ are maximal dissipative.

#### Proof

The identity (8.5) shows that $\langle A(v,\mathbf {w}),(v,{\mathbf {w}})\rangle _{{\mathcal {X}}}=0$ for all $(v,{\mathbf {w}})\in \mathrm {V}_h \times {\mathbf {W}}_h^0.$ Given $(f,{\mathbf {f}})\in \mathcal {X}$, solving the coercive problem

$$\begin{aligned} {\text {find}} \; v\in \mathrm {V}_h:\qquad (\nabla v,\nabla \tau )_{{\mathbb {R}}^d}+(v,\tau )_{{\mathbb {R}}^d} =(f,\tau )_{{\mathbb {R}}^d}-({\mathbf {f}},\nabla \tau )_{{\mathbb {R}}^d} \quad \forall \tau \in \mathrm {V}_h, \end{aligned}$$

and defining ${\mathbf {w}}=\nabla v+{\mathbf {f}}$, we have a pair $(v,{\mathbf {w}})\in \mathrm {V}_h \times {\mathbf {W}}_h^0$ such that $(v,{\mathbf {w}})-A(v,{\mathbf {w}})=(f,{\mathbf {f}})$ and thus *A* is maximal dissipative. The proof that $-A$ is maximal dissipative is similar. (Note that this is a particular case of what appears in [13].) $\square$

We consider the standard problem (2.1a–2.1c) with vanishing initial conditions and data $F=0$ and $\varXi =g:[0,\infty )\rightarrow L^2(\varGamma )$, namely, we look for $(v_h,{\mathbf {w}}_h):[0,\infty )\rightarrow {\text {dom}} \left( A_\star \right)$ such that

8.6a $$\begin{aligned}\begin{aligned}&(\dot{v}_h(t),\dot{{\mathbf {w}}}_h(t))= (\nabla \cdot {\mathbf {w}}_h(t),\nabla v_h(t))\quad\forall t>0, \end{aligned} \end{aligned}$$

8.6b $$\begin{aligned}\begin{aligned}&\big \langle \gamma _\nu ^+ {\mathbf {w}}_h(t)-\gamma _\nu ^- \mathbf {w}^h(t),\mu \big \rangle _{\varGamma }=\langle g(t),\mu \rangle_{\varGamma } \quad \forall \mu \in Y_h \quad \forall t>0, \end{aligned} \end{aligned}$$

8.6c $$\begin{aligned}&(v_h(0),{\mathbf {w}}_h(0))=(0,{\mathbf {0}}). \end{aligned}$$

Uniqueness of the solution to (8.6a–8.6c) follows from Proposition 8.2. We will handle existence of a solution below. The quantities of interest are $u_h:=\partial ^{-1}v_h$ and its Dirichlet trace $\psi _h:=\llbracket \gamma u_h \rrbracket :\; [0,\infty ) \rightarrow Y_h$.

#### Proposition 8.3

There exists a linear bounded right inverse ${\mathscr {E}}:{\mathcal {M}} \rightarrow {\text {dom}} \left( A_\star \right)$ of *B* such that $\mathrm {range}\,{\mathscr {E}}\subset \ker (I-A_\star )$. The norm of ${\mathscr {E}}$ is independent of the space $Y_h$.

#### Proof

Given $\xi \in {\mathcal {M}}=H^{-1/2}(\varGamma )$, we solve the coercive problem

8.7 $$\begin{aligned}& {\text {find }} v\in \mathrm V_h:\quad&(\nabla v,\nabla \tau )_{{\mathbb {R}}^d \setminus \varGamma }+(v,\tau )_{{\mathbb {R}}^d \setminus \varGamma }= \langle \xi , \gamma ^+ {\tau } \rangle _{\varGamma } \quad \forall \tau \in \mathrm V_h, \end{aligned}$$

and we set ${\mathbf {w}}:=\nabla v$.

This problem is equivalent to (note (8.5))

8.8 $$\begin{aligned} (v,{\mathbf {w}})\in {\text {dom}} \left( A_\star \right) , \qquad (v,\mathbf {w})=A_\star (v,{\mathbf {w}}), \qquad B(v,{\mathbf {w}})=\xi . \end{aligned}$$

Since $\left| \langle \xi , \gamma ^+ {w} \rangle _{\varGamma }\right| \lesssim \left\| \xi \right\| _{H^{-1/2}(\varGamma )} \left\| w\right\| _{H^1({\mathbb {R}}^d \setminus \varGamma )}$ it follows that the norm of the solution operator for (8.7) is independent of the space $Y_h$. $\square$

#### Proposition 8.4

The lifting ${\mathscr {E}}$ from Proposition 8.3 is a bounded linear map $L^2(\varGamma ) \rightarrow {\mathcal {X}}_{1/2}:=[\mathcal {X},{\text {dom}} (A)]_{1/2}$ with $\left\| {\mathscr {E}}\lambda \right\| _{{\mathcal {X}}_{1/2}} \le C \left\| \lambda \right\| _{L^2(\varGamma )}.$ The constant *C* depends only of $\varOmega$.

#### Proof

We will need spaces encoding homogeneous normal traces:

$$\begin{aligned} {\mathbf {H}}_0(\mathrm {div},\varOmega )&:=\big \{ {\varvec{w}} \in {\mathbf {H}}(\mathrm {div},\varOmega ): \gamma _{\nu }^- {\varvec{w}} =0\big \}, \\ {\mathbf {H}}_0(\mathrm {div},{\mathbb {R}}^d\setminus \overline{\varOmega }))&:=\big \{ {\varvec{w}} \in {\mathbf {H}}(\mathrm {div}, {\mathbb {R}}^d\setminus \overline{\varOmega })): \gamma _{\nu }^+ {\varvec{w}} =0\big \}. \end{aligned}$$

Let $\lambda \in L^2(\varGamma )$ be given. By applying Theorem A.4 to the exterior and setting $\widetilde{{\mathbf {w}}} =0$ inside, we can construct a function $\widetilde{{\mathbf {w}}} \in \mathbf {H}(\mathrm {div},{\mathbb {R}}^d\setminus \varGamma )$ satisfying $\llbracket \gamma _{\nu } \widetilde{{\mathbf {w}}}\rrbracket = \lambda$ and

8.9 $$\begin{aligned} \left\| \widetilde{{\mathbf {w}}}\right\| _{\mathbf {H}(\mathrm {div},{\mathbb {R}}^d \setminus \varGamma )}+ \left\| \widetilde{{\mathbf {w}}}\right\| _{[L^2(\varOmega ), \mathbf {H}_0(\mathrm {div},{\mathbb {R}}^d\setminus \overline{\varOmega })]_{1/2}} \lesssim \left\| \lambda \right\| _{L^2(\varGamma )}. \end{aligned}$$

Upon identifying the product of function spaces on $\varOmega$ and ${\mathbb {R}}^d \setminus \overline {\varOmega}$ with a function space on ${\mathbb {R}}^d \setminus \overline {\varGamma}$, we have

$$\begin{aligned} {\mathbf {H}}_0(\mathrm {div},{\varOmega }) \times \mathbf {H}_0(\mathrm {div},{\mathbb {R}}^d\setminus \overline{\varOmega }) \subseteq {{\varvec{W}}}_h^0. \end{aligned}$$

The product of interpolation spaces equals the interpolation of product spaces (cf. Lemma A.5); we can therefore also estimate:

$$\begin{aligned} \left\| (0,\widetilde{{\mathbf {w}}})\right\| _{{\mathcal {X}}_{1/2}} \lesssim \left\| \widetilde{{\mathbf {w}}}\right\| _{[L^2(\varOmega ), {\varvec{W}}_h^0]_{1/2}} \lesssim \left\| \lambda \right\| _{L^2(\varGamma )}. \end{aligned}$$

If we consider $(v,{\mathbf {w}}) := {\mathscr {E}}\lambda$, then $(v, {\mathbf {w}} - \widetilde{{\mathbf {w}}}) \in {\text {dom}}(A)$ by construction of the lifting. Thus we have

$$\begin{aligned} \left\| (v,{\mathbf {w}})\right\| _{{\mathcal {X}}_{1/2}}&\le \left\| (v,{\mathbf {w}} - \widetilde{{\mathbf {w}}})\right\| _{{\mathcal {X}}_{1/2}} + \left\| (0, \widetilde{{\mathbf {w}}})\right\| _{{\mathcal {X}}_{1/2}} \\&\le \left( \left\| v\right\| _{H^1({\mathbb {R}}^d \setminus \varGamma )} + \left\| {\mathbf {w}} - \widetilde{{\mathbf {w}}}\right\| _{H({\text {div}},{\mathbb {R}}^d\setminus \varGamma )}\right) + \left\| (0,\widetilde{{\mathbf {w}}})\right\| _{{\mathcal {X}}_{1/2}}. \end{aligned}$$

The continuity of ${\mathscr {E}}$ from Proposition 8.3 and (8.9) conclude the proof. $\square$

#### Proposition 8.5

If $g\in {\mathcal {C}}^2([0,\infty );H^{-1/2}(\varGamma ))$ satisfies $g(0)=\dot{g}(0)=0$, then (8.6a–8.6c) has a unique strong solution.

#### Proof

Thanks to Propositions 8.2 and 8.3, this problem fits in the abstract framework described in [13], which proves existence and uniqueness of solution to (8.6a–8.6c). $\square$

Propositions 8.2 and 8.3 have some consequences. First of all, Assumption 2.I holds. Secondly, assuming $g(t) \in L^2(\varGamma )$, any solution to (2.1a–2.1c) with the above data ($F=0$, $\varXi =g$) is in $\mathcal X_{1/2}$, and therefore, solutions to (8.6a–8.6c) take values in ${\mathcal {X}}_{1/2}$ as well. Finally, if $g\in \mathcal C^s([0,\infty ];L^2(\varGamma ))$ then ${\mathscr {E}}g\in \mathcal C^s([0,\infty ];{\mathcal {X}}_{1/2})$.

We also need a regularity result that allows us to bound time derivatives of the solution in terms of the data. The continuity condition for the $(s+2)$-nd derivative of *g* in Proposition 8.6 can be relaxed to local integrability, but then the norms on the right-hand side of (8.10) have to be modified.

#### Proposition 8.6

If $g\in {\mathcal {C}}^{s+2}([0,\infty );L^2(\varGamma ))$ satisfies $g^{(\ell )}(0)=0$ for $\ell \le s+1$, then the unique solution to (8.6a–8.6c) satisfies

- $$(v_h,{\mathbf {w}}_h)\in {\mathcal {C}}^{s+1}([0,\infty );{\mathcal {X}})$$,
- $$(v_h,{\mathbf {w}}_h)\in \mathcal C^{s}([0,\infty );{\mathcal {V}})$$ and $$(v_h,{\mathbf {w}}_h)\in \mathcal C^{s}([0,\infty );{\mathcal {X}}_{1/2})$$,
- For all $\ell \le s$, there exists *C*, independent of the choice of $Y_h$, such that for all $t\ge 0$
  8.10 $$\begin{aligned} \Vert (v_h^{(\ell )}(t),{\mathbf {w}}_h^{(\ell )}(t))\Vert _{{\mathcal {X}}_{1/2}} \le C\,t\,\sum _{j=\ell }^{\ell +2} \max _{\tau \le t} \Vert g^{(j)}(\tau )\Vert _{L^2(\varGamma )}. \end{aligned}$$

#### Proof

This result follows from [13, Theorem 3.1]. To see item (b), we note that $(v_h,{\mathbf {w}}_h)$ is constructed by writing

$$\begin{aligned} (v_h(t),{\mathbf {w}}_h(t))= (v^0_h(t),{\mathbf {w}}^0_h(t)) + {\mathscr {E}}g(t), \end{aligned}$$

with $(v^0_h(t),{\mathbf {w}}^0_h(t)) \in {\text {dom}}(A)$. The statement then follows from Proposition 8.4. $\square$

We now consider the RK approximation of (8.6a–8.6c) in a finite time interval [0, *T*], which provides pairs of stage values $(V^k_{h,n},{\mathbf {W}}^k_{h,n})\in {\mathcal {X}}^m$ and step approximations $(v^k_{h,n},{\mathbf {w}}^k_{h,n})\in {\mathcal {X}}$. We then define

8.11 $$\begin{aligned} \{ U^k_{h,n}\}=(\partial ^k)^{-1}\{ V^k_{h,n}\}, \qquad u^k_{h,n}=r(\infty )u^k_{h,n}+{\mathbf {b}}^\top {\mathcal {Q}}^{-1}U^k_{h,n}, \quad n\ge 0 \end{aligned}$$

with $u^k_{h,0}=0$ (see Lemma 2.6) and $\psi ^k_{h,n}:=\llbracket \gamma u^k_{h,n} \rrbracket$ .

#### Proposition 8.7

For sufficiently smooth *g*, with RK approximations using a method satisfying Assumption 2.II, and with $\alpha$ given by (6.1), for $nk\le T$ we have the estimates

8.12 $$\begin{aligned} \Vert u_h(t_n)-u^k_{h,n}\Vert _{L^2({\mathbb {R}}^d\setminus \varGamma )}&\le C \left(T^2 + \alpha \right) k^{\min \{q+3/2+\alpha ,p\}} \sum _{\ell =q}^{p+3} \max _{t\le T} \Vert g^{(\ell )}(t)\Vert _{L^2(\varGamma )}, \end{aligned}$$

and

8.13 $$\begin{aligned} \Vert u_h(t_n)-u^k_{h,n}\Vert _{H^1({\mathbb {R}}^d\setminus \varGamma )}+ \Vert \psi _h(t_n)-\psi ^k_{h,n}\Vert _{H^{1/2}(\varGamma )} \le C \left( T^2 + \alpha \right) k^{\min \{q+1/2+\alpha ,p\}} \sum _{\ell =q}^{p+3} \max _{t\le T} \Vert g^{(\ell )}(t)\Vert _{L^2(\varGamma )}. \end{aligned}$$

The constants depend on $\varGamma$ and the Runge–Kutta method, but do not depend on *T* or on the choice of $Y_h$.

#### Proof

We will use Theorems 3.4 as well as Propositions 3.1 and 3.2. We note that $\rho _k(T)\le 1$ by Lemma 7.1 and Proposition 8.2. Also, with the ${\mathscr {E}}$ operator of Proposition 8.3, we have

8.14 $$\begin{aligned} \Vert {\mathscr {E}}\varXi ^{(\ell )}\Vert _{{\mathcal {X}}_{1/2}}\le C \Vert g^{(\ell )}\Vert _{L^2(\varGamma )}, \end{aligned}$$

with *C* independent of $Y_h$. The bound (8.12) follows from Theorem 3.4, using (8.10) and (8.14) to estimate the right-hand side. The bound

8.15 $$\begin{aligned} \Vert \nabla u_h(t_n)-\nabla u^k_{h,n}\Vert _{L^2({\mathbb {R}}^d\setminus \varGamma )} &=\Vert {\mathbf {w}}_h(t_n)-{\mathbf {w}}^k_{h,n}\Vert _{L^2({\mathbb {R}}^d \setminus \varGamma )} \nonumber \\&\le C T^2 k^{\min \{q+1/2+\alpha ,p\}} \sum _{\ell =q+1}^{p+3} \max _{t\le T} \Vert g^{(\ell )}\Vert _{H^{1/2}(\varGamma )} \end{aligned}$$

follows from Propositions 3.1 and 3.2, using (8.10) for the estimate in terms of the data. The $H^1({\mathbb {R}}^d\setminus \varGamma )$ estimate (8.13) is then a direct consequence of (8.12) and (8.15). The estimate for $\psi _h-\psi _h^k$ follows from the standard trace theorem. $\square$

### Scattering

As it does not incur much difficulty, we cover both the exterior scattering problem, which is an exterior Neumann problem, as well as the interior Neumann problem. In order to do so, we define the domains $\varOmega ^+:={\mathbb {R}}^d \setminus \overline{\varOmega }$ and $\varOmega ^-:=\varOmega$ and distinguish the problems by adding the superscripts $+$ or − to the functions involved. We stay in the geometric setting of the previous section. Assume that ${\mathbf {d}}\in {\mathbb {R}}^d$ is a unit vector (direction of propagation) and that $c\in {\mathbb {R}}$ is such that $\varOmega \subset \{{\mathbf {x}}\in {\mathbb {R}}^d\,:\, \mathbf {x}\cdot {\mathbf {d}}> c\}$. Let $\phi :{\mathbb {R}}\rightarrow {\mathbb {R}}$ be a function such that $\phi (r)=0$ for all $r\ge c.$ The incident wave $u^{\mathrm {inc}}({\mathbf {x}},t):=\phi ({\mathbf {x}}\cdot {\mathbf {d}}-t)$ propagates in the direction ${\mathbf {d}}$ at unit speed and has not reached the scatterer given by $\varOmega$ at time $t=0$. The data for our problem will be the function $g:[0,T]\rightarrow L^2(\varGamma )$ given by $g(t):=-\partial ^{\pm }_\nu u^{inc}(\cdot ,t)$.

The problems under consideration are: Find $u^{\pm }:[0,T]\rightarrow H^1(\varOmega ^\pm )$ satisfying

$$\begin{aligned} \ddot{u}^{\pm }(t)=\varDelta u^{\pm }(t), \qquad u^{\pm }(0)=\dot{u}^{\pm }(0)=0, \qquad \partial ^{\pm }_\nu u^{\pm }(t)=g(t), \end{aligned}$$

so that $\partial ^{\pm }_\nu (u^{\pm }+u^{\mathrm {inc}})=0$. (Note that we can take the trace of the normal derivative of the incident wave, since it is locally smooth.) The exterior problem (posed on $\varOmega ^+$) is the classical sound soft scattering problem of the incident wave $u^{\mathrm inc}$.

A direct formulation for solving this problem is obtained by extending the function by zero to the complement of the domain of interest. That is, we solve:

8.16 $$\begin{aligned} \ddot{u}^{\pm }(t)&=\varDelta u^{\pm }(t) \text{ in } \varOmega ^{\pm }, \quad \llbracket \partial _{\nu } u^{\pm }(t)\rrbracket =g(t), \quad \partial _\nu ^{\mp } u^{\pm }(t)=0 \end{aligned}$$

with $u^{\pm }(0)=\dot{u}^{\pm }(0)=0.$ By imposing some additional hypotheses on the growth of *g* (which is needed to have a well-defined distributional Laplace transform), we can represent the solution to (8.16) as $u^{\pm }={\mp }S(\partial )g - D(\partial ) \psi ^{\pm }$, where $\psi ^{\pm }:=\llbracket \gamma u^{\pm } \rrbracket$. Note that, to be precise with the use of weak distributional definitions, all functions have to be extended by zero to $t<0$ (we say that they are causal) and the time interval is extended to infinity.

Taking the trace in this representation formula, the solution of (8.16) can be found by solving an equation for $\psi ^{\pm }$ and then postprocessing with the potential operators:

8.17 $$\begin{aligned} {W}(\partial )\psi ^{\pm }=(1/2 \pm K^t(\partial )) g, \qquad u^{\pm }={\mp } S(\partial ) g - {D}(\partial ) \psi ^{\pm }, \end{aligned}$$

and we still have that $\psi ^{\pm }=\llbracket \gamma u^{\pm } \rrbracket$.

For simplicity of notation, we will skip the indices ± for the different functions from now on. We can equivalently write (8.16) and the equivalent (8.17) by using the variables $v:=\dot{u}$ and ${\mathbf {w}}:=\nabla u$. We note that $u=\partial ^{-1} v$ and $\psi =\partial ^{-1} \llbracket \gamma v \rrbracket$. Here, $(v,{\mathbf {w}})$ solve (we restrict *t* to the interval [0, *T*] again)

$$\begin{aligned} \dot{v}(t)=\nabla \cdot {\mathbf {w}}(t), \quad \dot{{\mathbf {w}}}(t)=\nabla v(t), \quad \llbracket \gamma _{\nu } {\mathbf {w}}(t)\rrbracket =g(t), \quad v(0)=0, \quad {\mathbf {w}}(0)={\mathbf {0}}, \end{aligned}$$

that is, (8.6a–8.6c) with $Y_h=H^{1/2}(\varGamma )$.

For the discretization, we consider a finite dimensional space $Y_h$ and the Galerkin approximation to (8.17), so that we look for $\psi _h:\,{\mathbb {R}}\rightarrow X_h$ causal such that

8.18 $$\begin{aligned} \langle {W}(\partial )\psi _h, \mu \rangle _\varGamma&= \langle (1/2 \pm K^t(\partial )) g, \mu \rangle _\varGamma \quad \forall \mu \in Y_h, \nonumber \\ u_h&:={\mp } {S}(\partial )g - D(\partial ) \psi _h. \end{aligned}$$

The functions $v_h:={\dot{u}}_h$ and ${\mathbf {w}}_h:=\nabla u_h$ satisfy (8.6a–8.6c). The difference between the solutions of (8.16) and (8.18) can be studied by comparing the solutions to (8.6a–8.6c) when $Y_h=H^{1/2}(\varGamma )$ and when $Y_h$ is a finite dimensional space, see [13] for details. For our purposes, it is sufficient to note that we get quasi-optimal estimates for the discretization in space.

Discretization in time is performed by applying convolution quadrature to (8.18). The fully discrete solution reads

8.19 $$\begin{aligned} \langle {W}(\partial ^k)\varPsi _h, \mu \rangle _\varGamma&= \langle (1/2 \pm K^t(\partial ^k)) g, \mu \rangle _\varGamma \quad \forall \mu \in Y_h,\; \; \nonumber \\ U_h:&={\mp }\mathrm {S}^{\prime}(\partial ^k)g - D(\partial ^k) \varPsi _h. \end{aligned}$$

The approximations $\psi ^k_h$ and $u^k_h$ are then computed by the usual post-processing, i.e.,

$$\begin{aligned} \begin{aligned} \psi ^k_{h,0}&:=0, \qquad\psi ^k_{h,n+1}=r(\infty ) \psi ^k_{h,n} + {\mathbf {b}}^T {\mathcal {Q}}^{-1} \varPsi ^k_{h,n}, \\ u^k_{h,0}&:=0, \qquad u^k_{h,n+1} =r(\infty ) u^k_{h,n} + {\mathbf {b}}^T {\mathcal {Q}}^{-1} U^k_{h,n}. \end{aligned} \end{aligned}$$

#### Lemma 8.8

The sequences $u^k_h$ and $\psi _h^k$ computed via (8.19) coincide with the Runge–Kutta approximations to (8.6a–8.6c) and their traces respectively.

#### Proof

The details of the computation can be found in the appendix of [23]. The basic idea is to take the Z-transform and show that both approaches solve the matrix-valued Helmholtz problem (4.2a–4.2c). $\square$

This gives the following immediate corollary, representing an *a priori* bound for the fully discrete method:

#### Corollary 8.9

Let the assumptions of Proposition 8.7 hold. Then for $u_h$ and $\psi _h$, approximated using convolution quadrature, we can estimate:

8.20 $$\begin{aligned}\Vert u_h(t_n)-u^k_{h,n}\Vert _{H^1({\mathbb {R}}^d\setminus \varGamma )} &+ \Vert \psi _h(t_n)-\psi ^k_{h,n}\Vert _{H^{1/2}(\varGamma )} \\& \le C (1+T^2) k^{\min \{q+1/2+\alpha ,p\}} \sum _{\ell =q}^{p+3} \max _{t\le T} \Vert g^{(\ell )}(t)\Vert _{L^2(\varGamma )}. \end{aligned}$$

The constants depend on $\varGamma$ and the Runge–Kutta method, but do not depend on *T* or on the choice of $Y_h$.

#### Remark 8.10

There is another approach for analyzing convolution quadrature methods, which is based on estimates in the Laplace domain. It can be shown that the Neumann-to-Dirichlet map, realized by the boundary integral Eq. (8.19), satisfies a bound of the form

$$\begin{aligned} \left\| W(s)^{-1}(1/2 \pm K^t(s)) \widehat{g}\right\| _{H^{1/2}(\varGamma )}\lesssim \frac{\left| s\right| }{{\text {Re}}(s)} \left\| \widehat{g}\right\| _{H^{-1/2}(\varGamma )} \quad \forall \widehat{g} \in H^{-1/2}  (\varGamma), \end{aligned}$$

see [20, Appendix 2]. Applying the abstract theory of [2] then implies convergence rate $\min (q+1,p)$ for the boundary data $\psi _h$. Modifying their proof, one can also get for $\widehat{g} \in L^2(\varGamma )$ that

$$\begin{aligned} \left\| W(s)^{-1}(1/2 \pm K^t(s)) \widehat{g}\right\| _{H^{1/2}(\varGamma )}\lesssim \frac{\left| s\right| ^{1/2}}{{\text {Re}}(s)} \left\| \widehat{g} \right\| _{L^2(\varGamma )}, \end{aligned}$$

which would yield the same convergence rate as Corollary 8.20, but without insight into the dependence on the end-time *T*. $\blacksquare$

### Numerical example

**Fig. 1:**
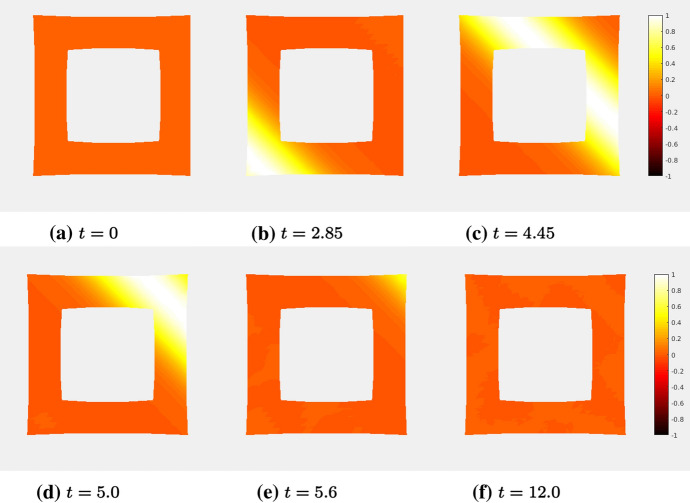
Snapshots of the simulation at $t=0$, $t=2.85$, $t=4.45$, $t=5.0$, $t=5.6$, $t=12$

We solve (8.19) on a “hollow square”, as depicted in Fig. 1, and focus on the interior Neumann problem, i.e. computing $\psi ^-=:\psi$. The geometry was chosen to be non-convex and not simply connected, in order to test if the rate observed is a general result, or if our estimates might prove sharp in some situation.

We prescribe the exact solution as a traveling wave, given by

$$\begin{aligned} u({\mathbf {x}}, t)&:=\phi ({\mathbf {x}} \cdot {\mathbf {d}} - t), \\ \phi (s)&:=\cos (\pi \,s/2) \, \exp (-4(s_0-s)^2). \end{aligned}$$

$s_0:=4$ is chosen so that $\phi (0)$ is sufficiently small in the domain. We set ${\mathbf {d}}:=[\frac{\sqrt{2}}{2},\frac{\sqrt{2}}{2}]^\top$ and solve up to an end time of $T=12$. An approximation of the $H^{1/2}$-error is computed via

$$\begin{aligned} \left\langle {W}(1) \left( \psi ^{k}_{h,n}- \varPi _{L^2} \psi (t_n)\right) , \psi ^{k}_{h,n}- \varPi _{L^2} \psi (t_n) \right\rangle _{\varGamma }, \end{aligned}$$

i.e., we compare to the $L^2$-projection of the exact solution. Since we are interested in the convergence rate with respect to the timestep size *k*, we consider a fixed, but sufficiently fine mesh.

We used 3 and 5 stage Radau IIA methods, with orders (*q*, *p*) of (3, 5) and (5, 9), respectively (see [16] for their definition). While their strong damping properties are not advantageous for wave propagation problems, they nevertheless are the standard method used with convolution quadrature. This is in part due to the fact that the standard theory (see, e.g., [2]) makes some assumptions not satisfied by the Gauss methods. A more detailed analysis of the dissipation and dispersion of the Radau methods was performed in [7, Sect. 4.3], showing that higher order Runge–Kutta methods posess favorable properties compared to their low order brethren.

**Fig. 2:**
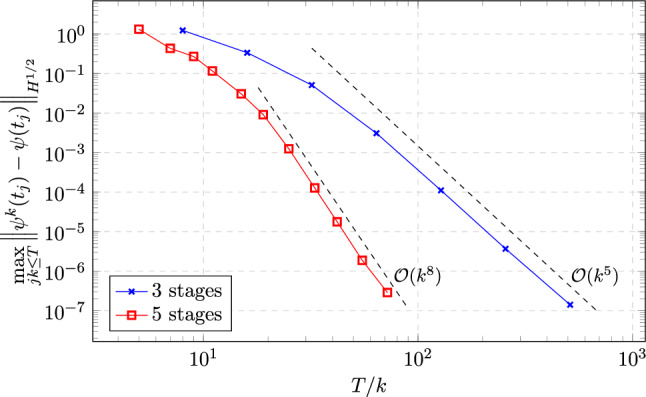
Performance of Radau IIA methods for the wave equation, cf. Sect. 8.4

Our theory predicts convergence rates of 4.5 and 6.5. In Fig. 2, we observe a rate that is closer to 5 and 8. This means that (just like the standard Laplace-domain estimates) our estimates do not appear to be sharp in this case. Further investigations into the cause of this phenomenon are required. Results trying to explain this phenomenon, initially prompted by the work on this article, can be found in [24] but with a different model problem.

### The heat equation

In this section, as an example where our estimates turn out to be sharp, we consider a heat conduction problem and will apply Theorem 3.5 to get convergence of the boundary trace. The physical situation is a body $\varOmega \subset {\mathbb {R}}^d$ that is held at a given temperature distribution and radiates heat into a medium $\varOmega ^+:={\mathbb {R}}^d \setminus \overline{\varOmega }$. We make the simplifying assumption that at $t=0$ the temperature is 0. Since the problem is posed on an unbounded domain, it is a good candidate for boundary integral equations, while being simple enough to showcase our more general results. We only briefly give the mathematical setting. More details and a more involved physical example can be found in [27]. The setting is as follows: find $u: {\mathbb {R}}_+ \rightarrow H^1_{\varDelta }(\varOmega ^+)$ such that

8.21a $$\begin{aligned} \begin{aligned} {\dot{u}} &= \varDelta u \quad \text {in } {\mathbb {R}}^d \setminus \overline{\varOmega }, \end{aligned} \end{aligned}$$

8.21b $$\begin{aligned} \begin{aligned} u(t)|_{\varGamma } &= g(t) \quad \text {on } \varGamma :=\partial \varOmega , \end{aligned} \end{aligned}$$

8.21c $$\begin{aligned}\begin{aligned} u(0) &= 0 \quad \text {in } {\mathbb {R}}^d \setminus \overline{\varOmega }. \end{aligned} \end{aligned}$$

It is well-known that $\varDelta$ with homogeneous Dirichlet boundary conditions generates an analytic semigroup (see e.g. [26, Sect. 7.2]) on $L^2({\mathbb {R}}^d \setminus \overline{\varOmega })$. The rest of our assumptions are also easily checked. We summarize:

(i) $${\text {dom}}(A_{\star })=\{u \in H^1({\mathbb {R}}^d \setminus \overline{\varOmega }): \varDelta u \in L^2({\mathbb {R}}^d \setminus \overline{\varOmega }) \}$$,
(ii) $B: H^1({\mathbb {R}}^d \setminus \overline{\varOmega }) \rightarrow H^{1/2}(\varGamma )=:{\mathcal {M}}, \; Bv := \gamma ^+ v$ (using the standard trace operator).

In order to derive the boundary integral formulation, we take the Laplace transform of (8.21a), giving for $\kappa :=\sqrt{s}$:

$$\begin{aligned} - \varDelta \widehat{u}(s) + \kappa ^2 \widehat{u}(s)&= 0, \end{aligned}$$

which is Helmholtz’s equation for a complex wave number $\kappa$. We make an ansatz of the form $\widehat{u}=S(\kappa ) \widehat{\lambda }$ for some unknown density $\widehat{\lambda }$, which can be determined by applying the trace operator, giving the equation $V(\kappa )\widehat{\lambda }={\mathscr {L}}(g)$.

Transforming back, and using the definition $V_{\kappa }(s):=V(\sqrt{s})$, we get the formulation:

$$\begin{aligned} \left[ V_{\kappa }(\partial ) \lambda \right] (t)&= g(t) \qquad \forall t>0. \end{aligned}$$

The solution *u* can then be recovered by computing $u=S_{\kappa }(\partial )$, where $S_\kappa (s):=S(\sqrt{s})$.

The discrete version of this is then given by solving

8.22 $$\begin{aligned} V_{\kappa }(\partial ^k) \varLambda ^k&= g. \end{aligned}$$

It can be shown that plugging the discrete solution into the representation formula $U^k:=S_{\kappa }(\partial ^k) \varLambda ^k$ gives back the Runge–Kutta approximation of (8.21a–8.21c). The approximations at the endpoints $t_n=n\,k$, denoted by $\lambda ^k$ and $u^k$ respectively can be computed by the usual post-processing. We refer to the appendix of [23] for an analogous computation in the context of the Schrödinger equation, which easily transfers to our situation. For simplicity, we do not consider any discretization in space. A Galerkin approach could easily be included into the analysis, analogously to Sect. 8.2.

From the definition $A:=A_{\star }|_{\ker (B)}$ we get ${\text {dom}}(A):= \big \{ u \in H^1({\mathbb {R}}^d \setminus \overline{\varOmega }): \varDelta u \in L^2({\mathbb {R}}^d \setminus \overline{\varOmega }), \; \gamma ^+ u =0 \big \}.$ We need the following analog of Proposition 8.4:

#### Proposition 8.11

$${\text {dom}}(A_{\star }) \subseteq [L^2({\mathbb {R}}^d \setminus \overline{\varOmega }), {\text {dom}}(A)]_{\mu ,\infty }$$ for $$\mu \in [0,1/4]$$.

#### Proof

It is easy to see that $H^2_0({\mathbb {R}}^d \setminus \overline{\varOmega }) \subseteq {\text {dom}}(A)$.

Using the Besov spaces introduced in Appendix A, we can write, if $\mu \le 1/4$:

$$\begin{aligned} H^1({\mathbb {R}}^d \setminus \overline{\varOmega })&\subseteq B^{2\mu }_{2,1}({\mathbb {R}}^d \setminus \overline{\varOmega }) {\mathop {\subseteq }\limits ^{Thm \;\; A.1}} \widetilde{B}^{2\mu }_{2,\infty }({\mathbb {R}}^d \setminus \overline{\varOmega }) \\&=[L^2({\mathbb {R}}^d \setminus \overline{\varOmega }), H_0^1({\mathbb {R}}^d \setminus \overline{\varOmega })]_{2\mu ,\infty } =[L^2({\mathbb {R}}^d \setminus \overline{\varOmega }), H_0^2({\mathbb {R}}^d \setminus \overline{\varOmega })]_{\mu ,\infty }, \end{aligned}$$

where in the last step, we used [22, Theorem B.9]. $\square$

The convergence of our numerical method can then be analyzed quite easily using Proposition 3.2 and Theorem 3.5.

#### Theorem 8.12

Let *p* and *q* denote the classical and stage order of the Runge–Kutta method used. Let $g \in {\mathcal {C}}^{p+3}([0,T], H^{1/2}(\varGamma ))$ with $g^{(j)}(0)=0$ for $j=0,\ldots , p+2$. Set $\alpha :=1$ if the Runge–Kutta method satisfies Assumption 2.III and $\alpha :=0$ otherwise. Then the following estimate holds for the post-processed approximation:

8.23 $$\begin{aligned} \left\| u^{k}(t_n) - u(t_n)\right\| _{L^2({\mathbb {R}}^d\setminus \overline{\varOmega })}&\le C (1+ T^2) k^{\min (q+\alpha +1/4,p)}\sum _{\ell =q+1}^{p+2} \max _{\tau \le t_{n}}{\left\| g^{(\ell )} ({\tau})\right\| _{H^{1/2}(\varGamma )}}. \end{aligned}$$

Assume that the Runge–Kutta method used for discretization is stiffly accurate. Then the following estimates hold for the $H^1$-norm:

8.24 $$\begin{aligned} \left\| u^{k}(t_n) - u(t_n)\right\| _{H^1({\mathbb {R}}^d\setminus \overline{\varOmega })}&\le C (1+T^2) k^{r_1}\sum _{\ell =q+1}^{p+3}\max _{\tau \le t_{n}}{\left\| g^{(\ell )} ({\tau})\right\| _{H^{1/2}(\varGamma )}}, \end{aligned}$$

with

$$\begin{aligned} r_1:={\left\{ \begin{array}{ll} q+ \alpha - 1/4 &{} \text { for } q< p-1, \\ q -1/4 &{} \text { for } q= p-1 \text { and }\alpha =0, \\ q+ 5/8 &{} \text { for } q= p-1 \text { and }\alpha =1,\\ q + \frac{\alpha -1}{2} &{} \text { for } q=p. \end{array}\right. } \end{aligned}$$

For the density, we get:

8.25 $$\begin{aligned} \left\| \lambda ^{k}(t_n) - \lambda (t_n)\right\| _{H^{-1/2}(\varGamma )}&\le C (1+T^2)\, k^{r_{\lambda }}\sum _{\ell =q}^{p+3}\max _{\tau \le t_{n}}{\left\| g^{(\ell )} ({\tau})\right\| _{H^{1/2}(\varGamma )}}, \end{aligned}$$

where the rate $r_{\lambda }$ is given by:

$$\begin{aligned} r_{\lambda }:={\left\{ \begin{array}{ll} q+ \alpha - 1/2 &{} \text { for } q< p-1, \\ q - 1/2 &{} \text { for } q= p-1 \text { and }\alpha =0, \\ q+ \frac{7}{16} &{} \text { for } q= p-1 \text { and }\alpha =1,\\ q + \frac{3}{4} (\alpha -1) &{} \text { for } q=p. \end{array}\right. } \end{aligned}$$

#### Proof

We first note that we can control the derivatives $u^{(\ell )}$ by the data. This can be done completely analogous to Proposition 8.6 by the techniques of [13]. The estimates read:

$$\begin{aligned} \Vert {u^{(\ell )}(t)}\Vert _{L^2({\mathbb {R}}^d \setminus \overline{\varOmega })}&\le C t \sum _{j=\ell }^{\ell +1}\max _{\tau \le t} \Vert {g^{(j)} (\tau)}\Vert _{H^{1/2}(\varGamma )}. \end{aligned}$$

For simplicity of notation, we only consider the case $q<p-1$. All the other cases follow analogously but giving different rates when applying the abstract theory. By Proposition 8.11, we can apply Propositions 3.1 or 3.2 with $\mu =1/4$, depending on whether we are in the setting $\alpha =0$ or $\alpha =1$. This gives estimate (8.23).

Applying Theorem 3.5, we get the following convergence in the graph norm of $A_{\star }$:

8.26 $$\begin{aligned} \Vert {\varDelta u^{k}(t_n) - \varDelta u(t_n)}\Vert _{L^2({\mathbb {R}}^d \setminus \overline{\varOmega })}&\le C (1+T^2) k^{q+\alpha -1+1/4}\sum _{\ell =q}^{p+3}\max _{\tau \le t_{n}}{\left\| g^{(\ell )} ({\tau})\right\| _{H^{1/2}(\varGamma )}}. \end{aligned}$$

Since for stiffly accurate RK-methods, $u^k$ also satisfies the boundary conditions (it is just the last entry of the stage vector) we get from the Dirichlet-boundary conditions that $\gamma ^+ u(t_n) = g(t_n) = \gamma ^+ u^k(t_n)$. Therefore, integration by parts and the Cauchy-Schwarz inequality give:

$$\begin{aligned}&\Vert {\nabla u^{k}(t_n) - \nabla u(t_n)}\Vert _{L^2({\mathbb {R}}^d \setminus \overline{\varOmega })}^2 =-\big ( \varDelta u^{k}(t_n) - \varDelta u(t_n), u^{k}(t_n) - u(t_n)\big )_{L^2({\mathbb {R}}^d \setminus \overline{\varOmega })} \\&\quad \le \Vert { \varDelta u^{k}(t_n) - \varDelta u(t_n)}\Vert _{L^2({\mathbb {R}}^d \setminus \overline{\varOmega })}\Vert {u^{k}(t_n) - u(t_n)}\Vert _{L^2({\mathbb {R}}^d \setminus \overline{\varOmega })}. \end{aligned}$$

Estimate (8.24) then follows from (8.23) and (8.26). For the estimate (8.25) of the density, we fix $\xi \in H^{1/2}(\varGamma )$, and let *v* denote a lifting to $H^1({\mathbb {R}}^d)$. We calculate

$$\begin{aligned} \left | \left \langle{\lambda -\lambda ^k,\xi }\right \rangle_{\varGamma }  \right |& = \left | \big (-\varDelta u + \varDelta u^k,v\big )_{L^2(\varOmega )} + \big (\nabla u - \nabla u^k,\nabla v\big )_{L^2({\mathbb {R}}^d \setminus \overline{\varOmega })}\right | \\&\le \big ( k^{1/2}\,\Vert {\varDelta u - \varDelta u^k}\Vert _{L^2(\varOmega )} + \Vert {\nabla u - \nabla u^k}\Vert _{L^2({\mathbb {R}}^d \setminus \overline{\varOmega })}\big ) \\&\quad \times \big (k^{-1/2} \Vert {v}\Vert _{L^2(\varOmega )} + \Vert {\nabla v}\Vert _{L^2({\mathbb {R}}^d \setminus \overline{\varOmega })}\big ). \end{aligned}$$

We are still free to choose the precise lifting *v*. Doing so as in [31, Proposition 2.5.1], we get

$$\begin{aligned}\inf \{ k^{-1/2}\Vert v\Vert _{L^2({\mathbb {R}}^d)}+\Vert \nabla v\Vert _{L^2({\mathbb {R}}^d)} : v\in H^1({\mathbb {R}}^d),\,\gamma v=\xi \} \lesssim \max \{1,k^{-1/4}\}\Vert \xi \Vert _{H^{1/2}(\varGamma )}. \end{aligned}$$

The result then follows from the previous estimates. $\square$

#### Remark 8.13

Note that in the cases $q=p-1$ with $\alpha =1$ and $q=p$ with $\alpha =0$, the rates $r_1$ and $r_{\lambda }$ in Theorem 8.12 are sharp from what can be extracted from Theorem 3.5 and Propositions 3.1 and 3.2. Nevertheless, we expect it to be possible to extract better rates from a more explicit investigation of these limiting cases.

#### Numerical example

In order to demonstrate that the estimate (8.25) is sharp, we consider a simple model problem. Following [34], we take $\varOmega$ to be the unit sphere and consider a right-hand side *g*(*x*, *t*) of the form

$$\begin{aligned} g(x,t):=\psi (t)Y^m_n(x), \end{aligned}$$

where $Y^m_n$ is the spherical harmonic of degree *n* and order *m*. It is well-known that the spherical harmonics are eigenfunctions of the pertinent boundary integral operators. Most notably for us, we have

$$\begin{aligned} V(s) Y^m_n&= \mu _n(s) Y^m_n \quad \text {with } \quad \mu _n(s):=-s \,j_n(i\,s) \,h^{(1)}_n(i\,s), \end{aligned}$$

where $j_n$ denotes the spherical Bessel functions and $h_n^{(1)}$ is the spherical Hankel function of the first kind. Due to this relation, solving (8.22) becomes a purely one dimensional problem, i.e., we can write $\lambda (x,t)={\widetilde{\lambda }}(t) Y^m_n(x)$ and the solution can be easily computed to very high accuracy. For our experiments we chose $n=2$.

**Fig. 3:**
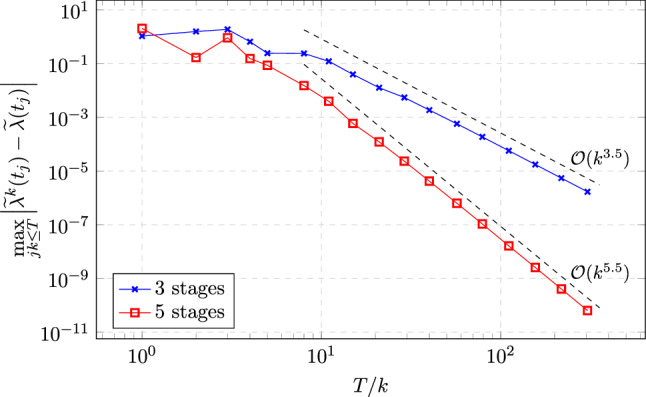
Convergence for the density ${\widetilde{\lambda }}$ for the heat conduction problem (cf. Sect. 8.5), comparing Radau IIA methods

We compare the 3-stage and 5-stage Radau IIA methods (see [16] for their definitions). These methods have stage orders 3 and 5 respectively and both are stiffly accurate and satisfy Assumption 2.III. We therefore expect convergence rates for the density $\lambda$ of order 3.5 and 5.5. Since the exact solution is not available, we compute the difference to an approximation with step size *k*/4 and use this as an approximation to the discretization error. The results can be seen in Fig. 3. We observe that the results are in good agreement with our predictions.

## A Interpolation of Sobolev spaces

In this appendix we prove that in Lipschitz domains and for certain parameters $\mu$, the spaces $[L^2(\varOmega ),H^1_0(\varOmega )]_{\mu }$ contain functions with non-vanishing boundary conditions. Such estimates are the main ingredient when determining the convergence rate of Runge–Kutta methods using the theory developed in the previous sections. For $\mu <1/2$, it is well-known that the fractional Sobolev spaces $H^\mu (\varOmega )=[L^2(\varOmega ),H^1(\varOmega )]_{\mu ,2}$ and $\widetilde{H}^{\mu }(\varOmega )=[L^2(\varOmega ),H^1_0(\varOmega )]_{\mu ,2}$ coincide (see e.g. [22, Theorem 3.40] together with the results in [22, Appendix B] to identify the Sobolev spaces with the interpolation space). We prove that when interpolating using the index $\infty$, the critical value $\mu =1/2$ is also admissible, provided that some further regularity is provided.

In order to state our result, we need additional notation, notably we define interpolation spaces for $q\in [1,\infty )$ as

A.1 $$\begin{aligned} \left\| u\right\| _{[{\mathcal {X}}_0,{\mathcal {X}}_1]_{\mu ,q}}^q&:=\int _{0}^{\infty }{t^{-\mu } \left[ \inf _{v \in {\mathcal {X}}_1} \left\| u-v\right\| _{{\mathcal {X}}_0} + t \left\| v\right\| _{{\mathcal {X}}_1}\right] ^q \frac{dt}{t}}, \end{aligned}$$

and introduce the following Besov spaces:

A.2 $$\begin{aligned} B^\mu _{2,q}(\varOmega ):=\left[ L^2(\varOmega ), H^1(\varOmega )\right] _{\mu ,q} \qquad \text {and}\qquad \widetilde{B}^\mu _{2,q}(\varOmega ):=\left[ L^2(\varOmega ), H^1_0(\varOmega )\right] _{\mu ,q}. \end{aligned}$$

For $t >0$, we define the strip

A.3 $$\begin{aligned} \varOmega _t:=\big \{x \in \varOmega : {\text {dist}}(x,\partial \varOmega ) < t\big \}, \end{aligned}$$

which will play an important role in the following proofs.

### Theorem A.1

Let $\varOmega$ be either a bounded Lipschitz domain or the complement of a bounded Lipschitz domain. Fix $\mu \in (0,1/2]$. Then

$$\begin{aligned} B^\mu _{2,1}(\varOmega )\subseteq \widetilde{B}^\mu _{2,\infty }(\varOmega ) \end{aligned}$$

with equivalent norms. The implied constant depends on $\varOmega$ and $\mu$.

### Proof

For simplicity, assume that $\varOmega$ is bounded. We focus on the case $\mu =1/2$, since the case $0 \le \mu < 1/2$ follows from $H^\mu (\varOmega ) = {\widetilde{H}}^\mu (\varOmega )$ (see [22, Theorem 3.40(i) and Theorem 3.33]) combined with the embeddings

$$\begin{aligned} B^\mu _{2,1}(\varOmega ) \subset B^\mu _{2,2}(\varOmega ) = H^\mu (\varOmega ) \subset {\widetilde{H}}^\mu (\varOmega ) \subset \widetilde{B}^\mu _{2,\infty }(\varOmega ). \end{aligned}$$

Consider $u \in B^{1/2}_{2,1}(\varOmega )$. For each $t>0$, we select $v(t) \in H^1(\varOmega )$ almost realizing the infimum appearing in the interpolation norm, i.e.,

$$\begin{aligned} \left\| u-v(t)\right\| _{L^2(\varOmega )} + t \left\| v(t)\right\| _{H^1(\varOmega )} \le 2 \inf _{w \in H^1(\varOmega )} \left( \left\| u-w\right\| _{L^2(\varOmega )} + t \left\| w\right\| _{H^1(\varOmega )}\right) . \end{aligned}$$

By [6, Lemma], the following estimate holds for all $t> 0$:

$$\begin{aligned} \left\| v(t)\right\| _{B^\mu _{2,1}(\varOmega )}\le 3 \left\| u\right\| _{B^{1/2}_{2,1}(\varOmega )}. \end{aligned}$$

We also note that

$$\begin{aligned} \Vert u - v(t)\Vert _{L^2(\varOmega )} + t \Vert v(t)\Vert _{H^1(\varOmega )} \lesssim t^{1/2} \Vert u\Vert _{[L^2(\varOmega ),H^1(\varOmega )]_{1/2,\infty }} \lesssim t^{1/2} \Vert u\Vert _{B^{1/2}_{2,\infty }(\varOmega )} \lesssim t^{1/2} \Vert u\Vert _{B^{1/2}_{2,1}(\varOmega )}. \end{aligned}$$

We consider a smooth cutoff function $\chi _t: \varOmega \rightarrow [0,1]$ satisfying:

A.4 $$\begin{aligned} \chi _t(x)&\equiv 0 \; \text { on }\varOmega _t, \qquad \chi _t(x) \equiv 1 \; \text { on }\varOmega \setminus \varOmega _{2t} \qquad \text { and } \quad \left\| \nabla \chi _t\right\| _{L^{\infty }} \lesssim t^{-1}. \end{aligned}$$

We then define $\widetilde{v}(t):=\chi _t v(t) \in H_0^1(\varOmega )$ and calculate:

$$\begin{aligned} \left\| u-\widetilde{v}(t)\right\| _{L^2(\varOmega )}&\le \left\| u-{v}(t)\right\| _{L^2(\varOmega )} + \left\| (1-\chi _t)v(t)\right\| _{L^2(\varOmega _{2t})}\\&\lesssim \left\| u-{v}(t)\right\| _{L^2(\varOmega )} + t^{1/2}\left\| v(t)\right\| _{B^{1/2}_{2,1}(\varOmega )} \end{aligned}$$

where we used the fact that $1-\chi _t$ vanishes on $\varOmega \setminus \varOmega _{2t}$ and applied [18, Lemma 2.1] to estimate the $L^2$-norm there.

Similarly,

$$\begin{aligned} t\,\left\| \widetilde{v}(t)\right\| _{H^1(\varOmega )}&\lesssim t\, \left\| v\right\| _{H^1(\varOmega )} + t\, \left\| (\nabla \chi _t) v(t)\right\| _{L^2(\varOmega )} \lesssim t\, \left\| v(t)\right\| _{H^1(\varOmega )} + \left\| v(t)\right\| _{L^2(\varOmega _{2t})} \\&\lesssim t \left\| v(t)\right\| _{H^1(\varOmega )} + t^{1/2} \left\| v(t)\right\| _{B^{1/2}_{2,1}(\varOmega )}. \end{aligned}$$

For the interpolation norm, we therefore get

$$\begin{aligned} \left\| u\right\| _{\widetilde{B}^{1/2}_{2,\infty }(\varOmega )}&\lesssim {\text { ess sup}}_{t>0}\!\Big [ t^{-1/2} \big (\!\left\| u-v(t)\right\| _{L^2(\varOmega )} \!+\! t \left\| v(t)\right\| _{H^1(\varOmega )} + t^{1/2} \left\| v(t)\right\| _{B^{1/2}_{2,1}(\varOmega )} \big )\Big ] \\&\lesssim \left\| v(t)\right\| _{B^{1/2}_{2,1}(\varOmega )} +\left\| u\right\| _{\left[ L^2(\varOmega ), H^1(\varOmega )\right] _{\mu ,\infty }} \lesssim \left\| u\right\| _{B^{1/2}_{2,1}(\varOmega )} +\left\| u\right\| _{B^{1/2}_{2,\infty }(\varOmega )} \\&\lesssim \left\| u\right\| _{B^{1/2}_{2,1}(\varOmega )}. \end{aligned}$$

If $\varOmega$ is the exterior of a bounded Lipschitz domain, the proof applies almost verbatim as all important steps can be localized to a neighborhood of the boundary. $\square$

### Remark A.2

The use of the second parameter $\infty$ in the interpolation norm is crucial for Theorem A.1 to hold in the case $\mu =1/2$. For $L^2$-based interpolation it is well-known that the interpolation space $\left[ L^2(\varOmega ), H^1_0(\varOmega )\right] _{1/2,2}$ is the Lions-Magenes space $H^{1/2}_{00}(\varOmega )$, see [35, Chapter 33], which is distinct from $H^{1/2}(\varOmega )$.

When considering the Neumann problem in Sect. 8.3, we need to devise a lifting to a vector field with a given normal jump in $L^2$. In general, such liftings do not have $B^{1/2}_{2,1}$-regularity. Thus Theorem A.1 is not applicable. Instead, we have a modified construction.

### Lemma A.3

Let $\varOmega$ be a bounded Lipschitz domain or the exterior of a bounded Lipschitz domain with boundary $\varGamma :=\partial \varOmega$. For $C > 0$, $c>0$ fixed with *c* sufficiently small, define the non-tangential maximal function

$$\begin{aligned} N(\nabla u)(x)&:=\!\!\sup _{y \in \varTheta (x)}{\left| \nabla u(y)\right| }, \; \text {where } \; \varTheta (x):=\{y \in \varOmega : \left| x-y\right| \le \max (c,C{\text {dist}}(y,\varGamma ))\}. \end{aligned}$$

Let $u \in H^1(\varOmega )$ be harmonic and satisfy $N(\nabla u) \in L^2(\varGamma )$.

Then for $t>0$ we can bound the $L^2$ norm on strips $\varOmega _t$ by

A.5 $$\begin{aligned} \left\| \nabla u\right\| _{L^2(\varOmega _t)}&\lesssim t^{1/2} \left\| N(\nabla u)\right\| _{L^2(\varGamma )}. \end{aligned}$$

### Proof

We focus on a single chart in the parametrization of (a vicinity of) $\varGamma$. Let ${\mathcal {O}} \subseteq \varOmega$ and ${\mathcal {D}} \subseteq {\mathbb {R}}^{d-1}$ be open, ${\varvec{r}} \in {\mathbb {R}}^n$, $\varphi : {\mathcal {D}}\rightarrow {\mathbb {R}}$, $y_0: {\mathcal {D}} \rightarrow {\mathbb {R}}$ such that we can write

$$\begin{aligned} \varOmega _t \cap {\mathcal {O}} = \big \{ (x, \varphi (x)+ y {\varvec{r}}): x \in {\mathcal {D}}, \text { and }y \in (0,y_0(x)) \big \}. \end{aligned}$$

By the Lipschitz assumption, we note that $y_0(x) \lesssim C t$. Following the considerations in [10, Appendix A.4], one can see that as long as *C* in the definition of $\varTheta$ is taken sufficiently large, we have that for all $x \in {\mathcal {D}}$

$$\begin{aligned} \{(x, \varphi (x)+ y {\varvec{r}}): y \in (0,y_0(x)) \} \subseteq \varTheta \bigl ((x, \varphi (x))\bigr ). \end{aligned}$$

We calculate

A.6 $$\begin{aligned} \left\| u\right\| _{L^2(\varOmega _t \cap {\mathcal {O}})}^2&=\int _{x \in {\mathcal {D}}}{ \int _{y=0}^{y_0(x)}{\left| \nabla u(x,\varphi (x)+y {\varvec{r}})\right| ^2 \,dy}\,dx} \nonumber \\&\lesssim \int _{x \in {\mathcal {D}}}{\int _{y=0}^{y_0(x)} {\big (N(\nabla u)(x)\big )^2 \,dy}\,dx} \nonumber \\&\le t\int _{x \in {\mathcal {D}}}{{\big (N(\nabla u)(x)\big )^2 \,dx} } \le t \left\| N(\nabla u)\right\| _{L^2(\varGamma )}^2. \end{aligned}$$

Repeating the same calculation for all boxes needed to parametrize a neighborhood of $\varGamma$ then concludes the proof. $\square$

### Theorem A.4

Let $\varOmega \subset {\mathbb {R}}^d$ be a bounded Lipschitz domain or the exterior of a bounded Lipschitz domain and write ${\mathbf {H}}_0(\mathrm {div},\varOmega ):=\{ {\varvec{w}} \in {\mathbf {H}}(\mathrm {div},\varOmega ): \gamma _{\nu } {\varvec{w}} =0\}$.

For every $g \in L^2(\varOmega )$, there exists a function ${\varvec{w}} \in {\mathbf {H}}(\mathrm {div},\varOmega )$ such that

A.7 $$\begin{aligned} \gamma _{\nu } {\varvec{w}}&=g \qquad \text {and} \qquad \left\| \nabla \cdot {\varvec{w}}\right\| _{H^1(\varOmega )}+\left\| {\varvec{w}}\right\| _{ [L^2(\varOmega ), {\mathbf {H}}_0(\mathrm {div},\varOmega )]_{1/2,\infty }} \lesssim \left\| g\right\| _{L^2(\varOmega )} \end{aligned}$$

with an implied constant depending only on $\varOmega$.

### Proof

For simplicity, assume that $\varOmega$ is bounded. By performing an appropriate cutoff away from $\partial \varOmega$, all arguments can be localized.

*Step 1:* Consider the case $\int _{\varGamma } g =0$. Let *u* be the solution of the Neumann problem

$$\begin{aligned} \varDelta u =0 \text { in }\varOmega , \qquad \partial _{\nu } u = g \text { on }\partial \varOmega , \qquad \int _{\varOmega } u = 0. \end{aligned}$$

In addition to $u \in H^1(\varOmega )$, by [17] (see also [10, Theorem A.6]), this harmonic function *u* also satisfies

$$\begin{aligned} \left\| N(\nabla u)\right\| _{L^2(\varGamma )} \le \left\| g\right\| _{L^2(\varGamma )}. \end{aligned}$$

For fixed $t>0$ we again select a smooth cutoff function $\chi _t$ satisfying (A.4). We set ${\varvec{w}}:=\nabla u$ and calculate using Lemma A.3:

$$\begin{aligned} \left\| {\varvec{w}}\right\| _{ [L^2(\varOmega ), \mathbf {H}_0(\mathrm {div},\varOmega )]_{1/2,\infty }}&\lesssim {\text { esssup}}_{t \ge 0}\Big ( t^{-1/2} \big [ \left\| (1-\chi _t) {\varvec{w}} \right\| _{L^2(\varOmega )} + t \left\| \chi _t {\varvec{w}} \right\| _{{\mathbf {H}}(\mathrm {div},\varOmega )} \big ]\Big ) \\&\lesssim {\text { esssup}}_{t \ge 0} t^{-1/2} \Vert \nabla u\Vert _{L^2(\varOmega _{2t})} \lesssim \left\| N(\nabla u)\right\| _{L^2(\varGamma )} {\lesssim }\left\| g\right\| _{L^2(\varGamma )}. \end{aligned}$$

*Step 2:* In the case $\int _{\varGamma } g \ne 0$, the harmonic Neumann problem does not have a solution. Instead, we define *u* as the solution to

$$\begin{aligned} -\varDelta u + u =0 \; \text {in }\varOmega , \qquad \partial _{\nu } u=g \; \text {on }\partial \varOmega , \end{aligned}$$

and again set ${\varvec{w}}:=\nabla u$. By construction we have $\nabla \cdot {\varvec{w}}=-u \in H^1(\varOmega )$.

We decompose $u=u_{0} + u_1$, where $u_0$ solves the full-space problem

$$\begin{aligned} -\varDelta u_0 = -u \text { in }{\mathbb {R}}^d \end{aligned}$$

where *u* was extended by 0 outside of $\varOmega$. As $u \in L^2({\mathbb {R}}^d)$, standard regularity theory gives $u_0 \in H^2(B)$ on any ball *B* and in particular $u_0 \in H^2(\varOmega )$. In turn this yields $\partial _{\nu } u_0 \in L^2(\partial \varOmega )$. By construction $u_1$ then solves

$$\begin{aligned} -\varDelta u_1 =0 \quad \text {in }\varOmega , \qquad \partial _{\nu} u_1=g - \partial _{\nu} u_0 \quad \text {on }\partial \varOmega . \end{aligned}$$

As *g*, $\partial _{\nu } u_0 \in L^2(\partial \varOmega )$ and $\int _\varGamma g - \partial _\nu u_0 = 0$, we can apply Step 1 to get

$$\begin{aligned} \nabla u_1 \in \big [L^2(\varOmega ), {\varvec{H}}_{0}({\text {div}},\varOmega )\big ]_{1/2,\infty }. \end{aligned}$$

Since $\nabla u_0 \in (H^1(\varOmega ))^d \subseteq (B^{1/2}_{2,1}(\varOmega ))^d$ we can apply Theorem A.1 to get

$$\begin{aligned} u_0 \in [(L^2(\varOmega ))^d, (H^1_0(\varOmega ))^d]_{1/2,\infty } \subset \bigl [L^2(\varOmega ), {\varvec{H}}_{0}({\text {div}},\varOmega )\bigr ]_{1/2,\infty } \end{aligned}$$

to conclude the proof of (A.7). $\square$

The following lemma appears to be known in the community, see e.g. [5, Section 3.13, Exercise 4], but in order to be able to rigorously cite, we provide a short proof.

### Lemma A.5

Let $X:=(X_1, \dots , X_N)$ and $Y:=(Y_1,\dots , Y_N)$, where $X_j, Y_j$ are Banach spaces with continuous embedding $Y_j \subset X_j$, and the product space carries any $l^{p}$-norm. Fix $q \in [1,\infty ]$ and $\theta \in (0,1).$ Then, the product of the interpolation spaces coincides with the interpolation of the product spaces. Namely, the following estimate holds for all $x:=(x_1,\dots ,x_N) \in [X,Y]_{\theta ,q}$:

$$\begin{aligned} N^{-1} \sum _{j=1}^{N}{\left\| x_j\right\| _{[X_j,Y_j]_{\theta ,q}}}&\le \left\| x\right\| _{[X,Y]_{\theta ,q}} \le \sum _{j=1}^{N}{\left\| x_j\right\| _{[X_j,Y_j]_{\theta ,q}}}. \end{aligned}$$

### Proof

For $j \in \{1,\dots ,N\}$, consider the operators $S_j$ and $T_j$ defined as

$$\begin{aligned} \begin{aligned} T_j: X&\rightarrow X_j \\ (x_1,\dots ,x_N)&\mapsto x_j \end{aligned} \qquad \text {and}\qquad \begin{aligned} S_j: X_j&\rightarrow X \\ x_j&\mapsto (0, \dots ,x_j,\dots 0). \end{aligned} \end{aligned}$$

It is easy to see using the interpolation estimate (2.6) that

$$\begin{aligned} \left\| T_j x\right\| _{[X_j,Y_j]_{\theta ,q}} \le \left\| x\right\| _{[X,Y]_{\theta ,q}} \quad \text {and} \quad \left\| S_j x_j\right\| _{[X,Y]_{\theta ,q}} \le \left\| x_j\right\| _{[X_j,Y_j]_{\theta ,q}}. \end{aligned}$$

We therefore calculate:

$$\begin{aligned} \sum _{j=1}^{N}{\left\| x_j\right\| _{[X_j,Y_j]_{\theta ,q}}}&= \sum _{j=1}^{N}{\left\| T_j x\right\| _{[X_j,Y_j]_{\theta ,q}}} \le \sum _{j=1}^{N}{\left\| x\right\| _{[X,Y]_{\theta ,q}}} \le N \left\| x\right\| _{[X,Y]_{\theta ,q}}. \end{aligned}$$

For the opposite direction, we observe that

$$\begin{aligned} \left\| x\right\| _{[X,Y]_{\theta ,q}}&=\big \Vert \sum _{j=1}^{N}{S_j x_j}\big \Vert _{[X,Y]_{\theta ,q}} \le \sum _{j=1}^{N}{\left\| S_j x_j\right\| _{[X,Y]_{\theta ,q}}} \le \sum _{j=1}^{N}{\left\| x_j\right\| _{[X_j,Y_j]_{\theta ,q}}}. \end{aligned} \quad {\square}$$
